# Microtubules as Platforms for Assaying Actin Polymerization In Vivo

**DOI:** 10.1371/journal.pone.0019931

**Published:** 2011-05-16

**Authors:** J. Margit Oelkers, Marlene Vinzenz, Maria Nemethova, Sonja Jacob, Frank P. L. Lai, Jennifer Block, Malgorzata Szczodrak, Eugen Kerkhoff, Steffen Backert, Kai Schlüter, Theresia E. B. Stradal, J. Victor Small, Stefan A. Koestler, Klemens Rottner

**Affiliations:** 1 Helmholtz Centre for Infection Research, Braunschweig, Germany; 2 Institute of Molecular Biotechnology, Austrian Academy of Sciences, Vienna, Austria; 3 Department of Developmental and Regenerative Biology, Institute of Medical Biology, Immunos, Singapore, Singapore; 4 Molecular Cell Biology Laboratory, Department of Neurology, Bavarian Genome Research Network, University Hospital Regensburg, Regensburg, Germany; 5 School of Biomolecular and Biomedical Sciences, University College Dublin, Dublin, Ireland; 6 Institute for Molecular Cell Biology, University of Münster, Münster, Germany; 7 Institute of Genetics, University of Bonn, Bonn, Germany; Swiss Federal Institute of Technology Zurich, Switzerland

## Abstract

The actin cytoskeleton is continuously remodeled through cycles of actin filament assembly and disassembly. Filaments are born through nucleation and shaped into supramolecular structures with various essential functions. These range from contractile and protrusive assemblies in muscle and non-muscle cells to actin filament comets propelling vesicles or pathogens through the cytosol. Although nucleation has been extensively studied using purified proteins in vitro, dissection of the process in cells is complicated by the abundance and molecular complexity of actin filament arrays. We here describe the ectopic nucleation of actin filaments on the surface of microtubules, free of endogenous actin and interfering membrane or lipid. All major mechanisms of actin filament nucleation were recapitulated, including filament assembly induced by Arp2/3 complex, formin and Spir. This novel approach allows systematic dissection of actin nucleation in the cytosol of live cells, its genetic re-engineering as well as screening for new modifiers of the process.

## Introduction

The actin cytoskeleton in higher eukaryotes comprises numerous sub-compartments, the molecular constituents and regulation of which are just beginning to be elucidated. Examples include protrusive organelles, such as lamellipodia and filopodia in migrating cells, adhesive and invasive structures including focal adhesions, the immunological synapse and invadosomes [1], [2] and also sensory structures, such as dendritic spines [3], [4]. Through its ability to form helical polar rods by polymerization, actin constitutes a versatile building unit for both pushing and pulling (in concert with myosin). The starting point for actin filament assembly is the formation of a nucleus of three actin monomers, which is considered to constitute the rate-limiting step *in vitro* and *in vivo*. Once actin filaments are nucleated, other accessory proteins are thought to take over and promote their elongation at their fast-growing ends. Whether this is accomplished by the nucleator or another factor depends on the mechanism of nucleation (see also below). Due to head to tail assembly of actin monomers, actin filaments are intrinsically polar, harboring a fastgrowing, barbed and a slowly growing, pointed end. The differential critical concentrations of polymerization at the two ends can cause net flow of actin monomers through the filament in a process known as treadmilling [5],[6], also operating in cellular structures like the lamellipodium [7], [8]. In addition, filament ends and sides are subject to regulation by uncountable filament binding factors with numerous activities. These include e.g. capping, stopping and protecting ends from growth and depolymerization, respectively, severing, generating filament ends presumably prone to disassembly, or bundling, explicitly amplified in contractile structures or finger-like protrusions such as filopodia and microvilli. It is commonly agreed that a composite of all these activities drives the turnover of a complex structure such as the lamellipodium. Nevertheless, it is also clear that the essential prerequisite of formation and maintenance of a given actin structure, lamellipodium or smallish actin accumulation accompanying endocytosis, constitutes the nucleation event, and an impressive progress has recently been made in the discovery of mechanisms and factors catalyzing this important process. Today, actin filament nucleators are roughly divided into three classes [9]: those that mimic a nucleus, like Arp2/3 complex, those that stabilize spontaneously formed intermediates, like formins, and those that recruit and align actin monomers, like Spir. Arp2/3 complex contains two actin-related proteins, Arp2 and Arp3 that, together with an actin monomer, can assemble into a nucleus ready for elongation, either individually or in association with a socalled mother filament [10]. In either case, Arp2/3 complex will stay attached to and protect perhaps the pointed end from depolymerization, but the barbed end will be free for monomer addition.

However, Arp2/3 complex is inactive in the absence of nucleation promoting factors (NPFs), which can deliver actin monomers and promote nucleation by inducing a conformational change in the complex considered to bring Arp2 and Arp3 in close proximity to each other [11]. It is assumed today that the multiple activities of the complex observed *in vivo* are mostly regulated by the continuously growing group of NPFs, which now appear to execute quite distinct and complementary functions determined e.g. by differential localization [11], [12], [13]. NPFs are grouped into two types. WASP family proteins constitute the type I NPFs like the name-giving Wiskott-Aldrich-Syndrome protein (WASP), N-WASP or Scar/WAVE proteins, all of which share at their C-termini modules for binding actin and Arp2/3 complex [14]. This domain formerly known as WA comprises different numbers of actin monomer-binding domains, also called V (for verprolin-homology) or W (WASP homology 2) and a C-terminal CA domain (connector and acidic) which activates Arp2/3 complex [11]. So in case of N-WASP, the C-terminus mediating Arp2/3 complex-dependent actin nucleation used in this study is composed of two V-modules linked to the CA-domain (VVCA). The second group of Arp2/3 complex activators is known as type II NPFs and comprises in mammals the Src-kinase substrate cortactin and HS1 (haematopoietic-specific 1), the latter of which is expressed in cells of the immune system. At variance to type I NPFs, these proteins bind to actin filaments instead of monomers via a central repeat domain and the Arp2/3 complex through an N-terminal acidic region. Importantly, cortactin and HS1 appear to activate Arp2/3 complex less potently *in vitro* than type I NPFs [15].

As opposed to Arp2/3 complex, formin dimers can nucleate and elongate actin filaments by surfing on their barbed ends in a process also known as leaky capping [16]. Formins are large proteins, harboring numerous regulatory domains at their N-termini, and as business end the actin-binding FH2- (formin homology 2-) domain, frequently aided by an FH1- (formin homology 1-) module, thought to operate in delivering actin to FH2 through profilin-actin recruitment [16], [17]. The subgroup of Diaphanous-related formins (DRFs) is activated in a signal-dependent fashion to promote actin assembly upon release of autoinhibitory interactions between the DID- (Diaphanous inhibitory-) and the DAD- (Diaphanous autoregulatory-) domain at the very C-terminus. The class of proteins potentiating nucleation by scaffolding actin monomers, such as Spir [18] or Cobl [19] or Leiomodin in muscle [20] is still growing [21], [22], and considered to stay attached to the pointed end upon nucleation while the barbed end continues to grow. These nucleators usually employ three or four actin monomer-binding domains in one protein, as in case of three and four WH2-domains in Cobl [19] and Spir [18], respectively. However, additional studies e.g. on Spir indicate more complex biochemical activities and potential cooperations with other nucleators such as formins [23], [24], [25], [26], so precise mechanistic understanding of nucleation by these factors and their subsequent fate will require future investigation.

Nevertheless, considerable knowledge has already been obtained about the mode of function and regulation by accessory proteins of all these factors, especially *in vitro* and in purified conditions, but understanding their relevance *in vivo* is more challenging for numerous reasons. These include the plethora of unknown factors potentially interfering with straight forward interpretation of results received upon inhibition of a given factor, but also the fact that structures dependent on distinct nucleators like to intermingle with each other (e.g. lamellipodia and filopodia) within a given cellular compartment [2], [27], complicating analyses and faithful, objective interpretation. Finally, it is almost impossible to directly compare the potencies of different nucleators in physiologic actin structures, since cells perfectly tune the engagement of nucleators in specific structures, both qualitatively and quantitatively, so traditional functional interference with a nucleator will provide information on its relevance for a given structure, but not relative to another nucleator.

To circumvent this problem, and to directly examine the ability of a given factor to drive actin filament nucleation *in vivo*, we developed a novel assay in which the putative nucleator is targeted to the sides of microtubules. Microtubules belong to those subcellular structures that are essentially free of endogenous actin, and they hardly associate with potentially interfering, cellular membranes along their entire length. Moreover, microtubules offer a homogenous topology and comparable stability, allowing not only the detection of actin recruitment in fixed and live cells, but also the analysis of actin turnover by FRAP (fluorescence recovery after photobleaching) approaches. Finally, they are easily relocated in the electron microscope and can thus potentially be employed to study *in vivo* actin filament arrangements seeded by different combinations of nucleators and/or accessory factors.

## Results

### Targeting actin assembly to microtubules

**Figure 1:**
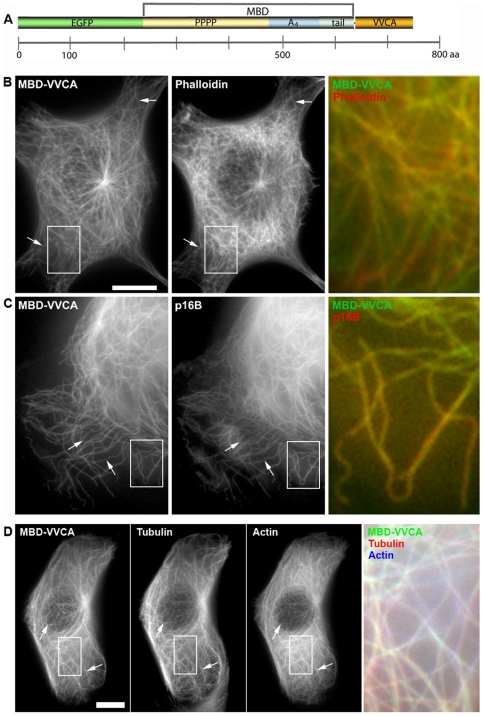
MBD-VVCA induces actin polymerization on microtubules by recruiting the Arp2/3 complex. (**A**) Domain structure of the EGFP-MBD-VVCA construct (MBD-VVCA). The EGFP-tag is fused to the microtubule-binding C-terminus of human MAP4 (MBD) and the VVCA-domain of N-WASP. (**B**) Phalloidin staining of a B16-F1 cell (red in merge) transiently expressing MBD-VVCA (green in merge). (**C**) B16-F1 cell co-transfected with MBD-VVCA (green in merge) and mCherry-tagged p16B to mark Arp2/3 complex (red in merge). Arrows denote co-localization of MBD-VVCA with actin (B) or Arp2/3 complex (C). Merges correspond to boxed insets in left panels. Bar, 10 µm. (**D**) Immunolabeling using anti-α-tubulin antibodies (red in merge) of a cell co-expressing EGFP-MBD-VVCA (green in merge) and EBFP-actin (blue in merge) to show co-localization of microtubules with the MBD-construct and actin. Merge corresponds to region boxed on the left. Bar,10 µm.

To test if actin assembly can be targeted to microtubules, we engineered a construct (pEGFP-MBD-VVCA, Figure 1A) encoding EGFP followed by a microtubule-binding sequence (MBD), and the VVCA-domain of murine N-WASP, which drives Arp2/3 complex-dependent actin nucleation [28]. The MBD harbors two independent microtubule-binding activities [29] located within the C-terminus of human MAP4 comprising a proline-rich, a so-called A_4_ domain and a tail region (see Methods). The EGFP-tagged MBD-VVCA-fusion targeted to microtubules and induced the assembly of actin filaments (F-actin), as evidenced by counter-staining with phalloidin (Figure 1B). Actin accumulation on microtubules was also detectable by live cell imaging (Figure S1A, Movie S1), and coincided with recruitment of Arp2/3 complex, visualized with mCherry-tagged p16B (also known as ArpC5B), a ubiquitously expressed isoform [30] of the smallest out of seven Arp2/3 complex subunits (Figure 1C, Movie S2). Among all subunits, fusions to p16 proved most useful for following Arp2/3 complex dynamics in mammalian cells [31], due perhaps to its peripheral location in the complex [10]. Importantly, MBD-VVCA-labeled structures strongly overlapped with both microtubules and actin, as evidenced by counter-staining of EGFP-MBD-VVCA and EBFP2-actin co-expressors with anti-tubulin antibodies (Figure 1D). Moreover, actin and Arp2/3 complex accumulation was specific for the presence of VVCA, since an identical construct lacking this domain (MBD) failed to stimulate actin assembly and Arp2/3 complex recruitment, in spite of its strong accumulation on microtubules (Figure S1B, Movie S3 and Movie S4). Conversely, EGFP-tagged VVCA alone failed to induce actin assembly on microtubules, since in the absence of a targeting domain it was unable to direct Arp2/3 complex activation to the microtubule surface (Figure S1C). Finally, western blotting confirmed expression of EGFP-tagged MBD or MBD-VVCA as full-length proteins, since no degradation pattern was observed using anti-GFP antibodies (Figure S1D).

**Figure 2:**
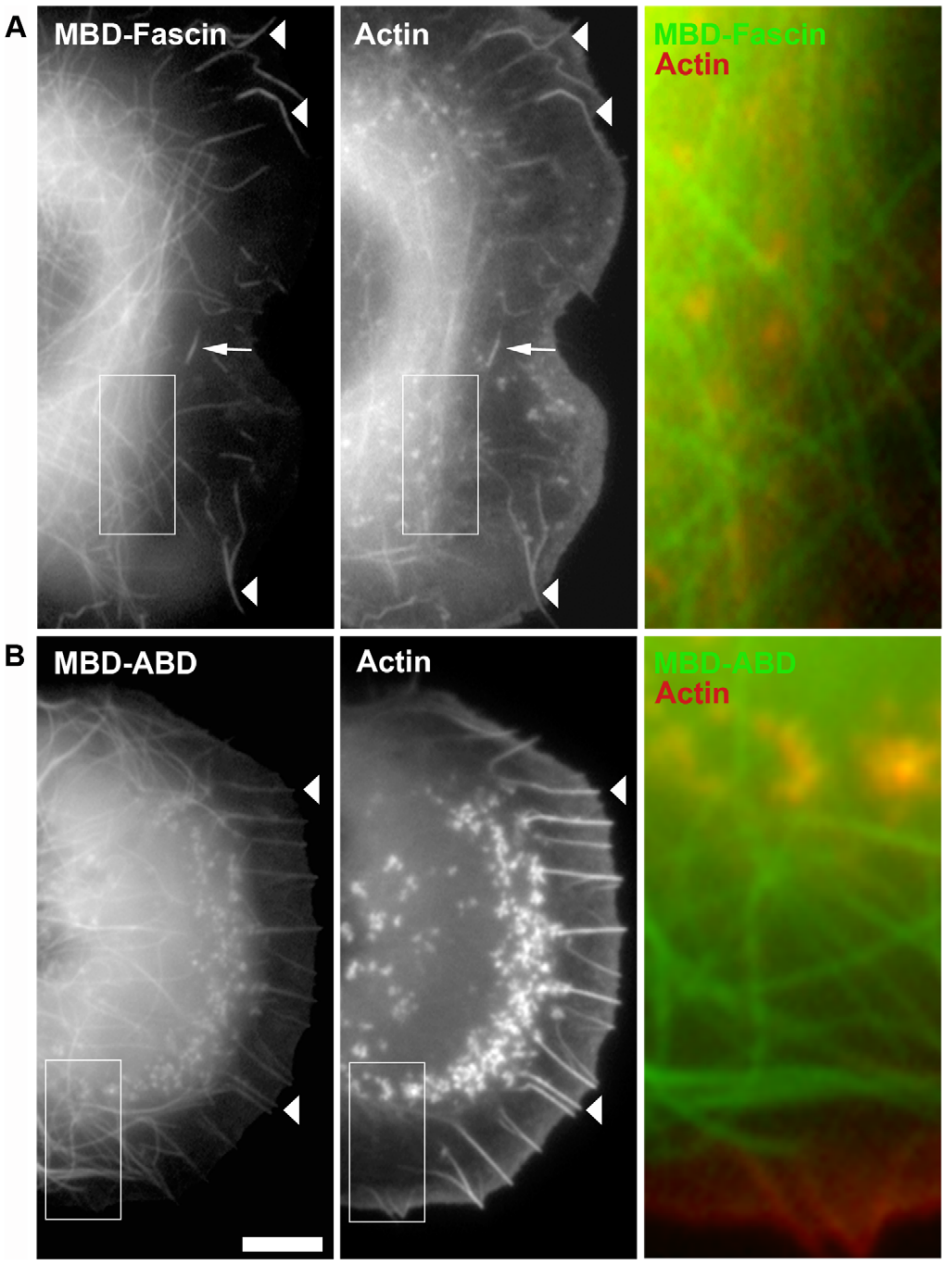
Actin filament binding is not sufficient to target actin to microtubules. Epifluorescence images of live cells co-expressing mCherry-actin with (**A**) EGFP-MBD-fascin or (**B**) EGFP-MBD-ABD (ABD: actin-binding domain of α-actinin). Arrowheads mark accumulation of MBD-fascin and MBD-ABD in microspikes embedded into the lamellipodium, as expected. Arrow points to a former microspike that became integrated into the lamella, as evidenced by video microscopy (data not shown). Merges (right) corresponding to boxed insets (left) reveal the absence of co-localization of these MBD-constructs with actin on microtubules. Bar, 5 µm.

It is thinkable that actin filaments are not nucleated on microtubules, but recruited instead as pre-nucleated, small filaments. Interestingly, the annealing of small actin oligomers has recently been proposed to contribute to actin assembly in yeast cells [32]. In addition, the two consecutive WH2-domains (VV) of N-WASP-VVCA were also demonstrated recently to interact with the barbed ends of actin filaments [33]. However, the following observations argue against oligomer recruitment to significantly contribute to actin assembly in our system. First, a truncated VVCA-domain lacking the C-terminus but comprising the two WH2-domains (VV) failed to recruit actin and Arp2/3 complex (Figure S2 and Figure S10, Movie S5 and Movie S6). The lack of Arp2/3 recruitment under these conditions emphasizes the need for physical interaction between the CA fragment and the Arp2/3 complex (see also below). Furthermore, to test whether an actin filament binding protein is capable of actin recruitment to microtubules, we produced chimeras of fascin and the actin-binding domain of α-actinin with MBD (Figure 2). Fascin is a prominent actin filament bundling protein, most famous for its strong association with microspikes and filopodia [34], which it serves to stabilize [35]. α-actinins are a family of dimeric actin-binding proteins with essential functions e.g. in signaling and stabilization of the contractile apparatus in muscle and analogous structures in non-muscle cells [36]. The fusion of EGFP-tagged MBD with fascin displayed a dual specificity pattern *in vivo* (Figure 2A): the MBD mediated microtubule localization, while fascin also targeted the fusion protein to microspike bundles at the cell periphery, as expected [35], [37]. Importantly, only the latter structures co-localized with actin, whereas MBD-fascin on microtubules did not show any sign of actin recruitment. Since MBD-fascin was expressed as a full-length protein (Figure S3B) capable of targeting to microspikes, the lack of actin accumulation on microtubules is unlikely due to non-functional protein folding. We also cloned the actin-binding domain (ABD) of α-actinin, which when tagged to EGFP alone labeled the actin cytoskeleton (Figure S3A). In contrast, a chimera of MBD and ABD of α-actinin (Figure 2B) again targeted to microspike bundles at the cell periphery and to microtubules, but there was no actin accumulation on the latter. Thus, we conclude that targeting of an actin-binding activity to microtubules potentially capable of recruiting small filaments or oligomers is not sufficient to drive actin assembly in this assay. Together with the lack of actin accumulation induced by N-WASP-VV, these data strongly suggest that VVCA-induced actin assembly on microtubules is mediated by *de novo* nucleation of actin filaments through the Arp2/3 complex.

### Topology of actin filaments assembled on microtubules

**Figure 3:**
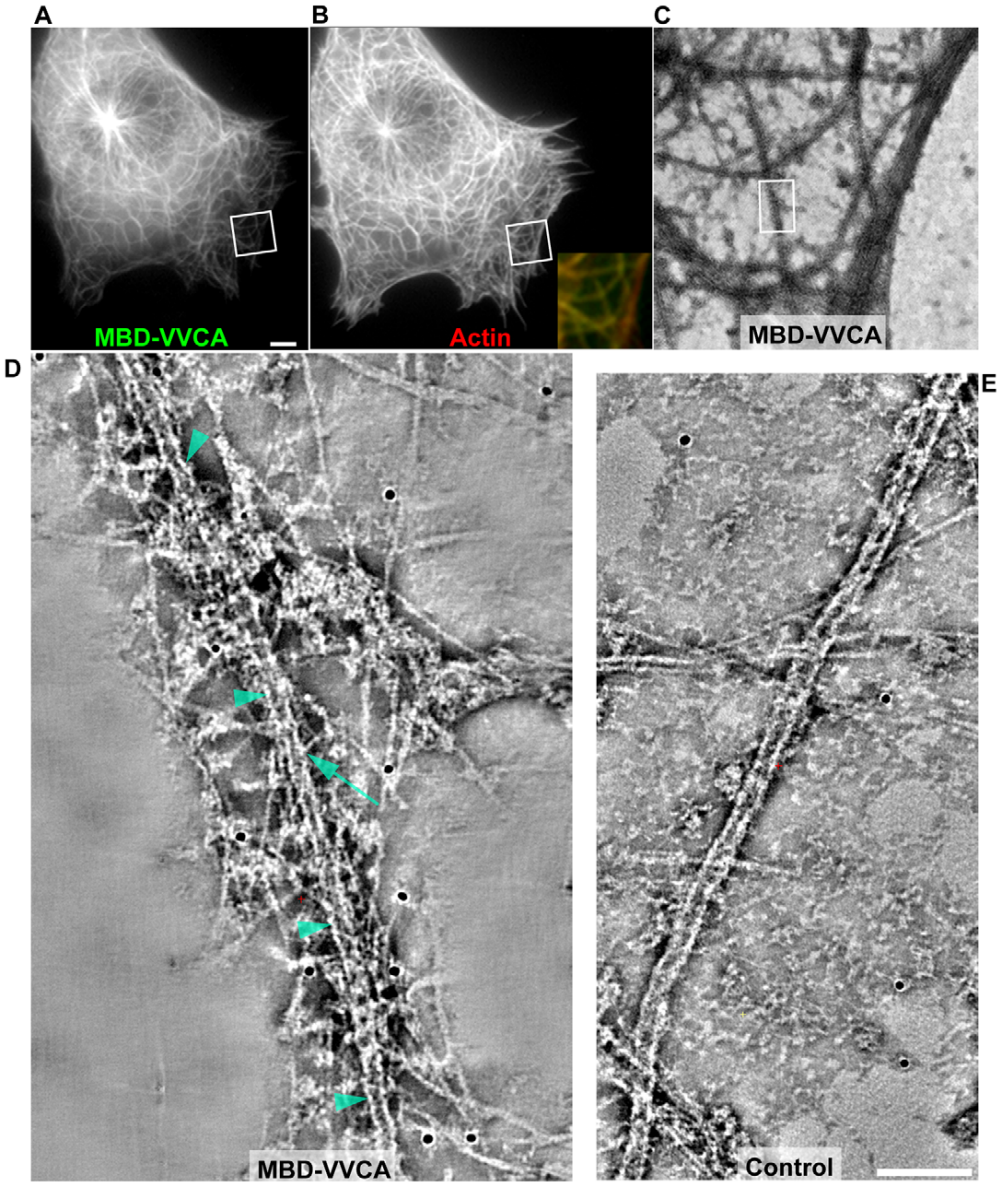
Ultrastructural arrangement of actin filaments induced on microtubules. (**A–D**) Correlated light microscopy and electron tomography of EGFP-MBD-VVCA/mCherry-actin double-transfected B16-F1 cell. (**A, B**). EGFP-MBD-VVCA (A) and mCherry-actin pattern (B) in B16-F1 cell after fixation on the light microscope. Inset in (B) shows merge of the regions boxed in (A) and (B); EGFP-MBD-VVCA is in green and mCherry-actin red, as indicated. Bar in (A) corresponds to 10 µm. (**C**) Overview electron microscopy image of the same cell after negative staining. Overview corresponds to merged inset in (B). (**D**) Composite image assembled from 30 slices (0.75 nm) of a tomogram of the region boxed in (C). Arrowheads in (D) mark surface of a single microtubule. Note the high density and disorganized arrangement of actin filaments located in close proximity to the microtubule (D), as compared to a microtubule in non-transfected control cell (E). Arrow marks an actin filament apparently attached to the microtubule surface. (**E**) Composite image assembled from 40 slices (0.75 nm) of a tomogram of an untransfected B16 cell, showing a microtubule traversing the field from the bottom left to the top right. Bar in (E) is valid for (D) and (E) and corresponds to 100 nm.

To assess the arrangement of Arp2/3 complex-induced actin filaments associated with microtubules, we performed correlative light microscopy and negative stain electron tomography. Control, non-transfected cells showed no specific association of actin filaments with microtubules, as expected (for representative microtubule see Figure 3E). Co-expression of mCherry-actin and EGFP-MBD-VVCA allowed confirmation of actin assembly on microtubules by observation in the light microscope (Figure 3A, B), and subsequent processing of the same cells by negative staining and electron microscopy. Individual microtubules labeled for MBD-VVCA and actin filaments in the light microscope were relocated in the electron microscope (Figure 3C). As shown, short actin filaments were found arranged as a disorganized cloud concentrated around microtubules, consistent with stochastic nucleation of actin filaments at the microtubule surface (Figure 3D). Individual actin filaments or filament stubs in physical contact with the microtubule surface could also be discerned (Figure 3D), indicative of at least transient direct interactions of nucleated actin filaments with microtubules, and also consistent with results obtained by FRAP (see below). These data confirm that microtubules can be exploited as platforms for actin nucleation *in vivo*, and can generate actin assemblies analyzable in detail by different types of light and electron microscopy.

### Arp2/3 dependent actin nucleation on microtubules is instantaneous and continuous

**Figure 4:**
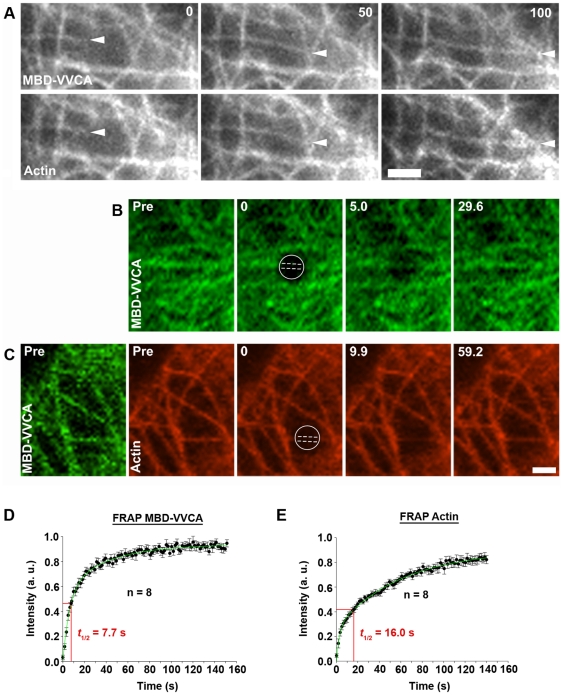
Turnover of MBD-VVCA and associated actin filaments on microtubules. (**A**) Selected frames derived from time-lapse movie of B16-F1 cell expressing EGFP-MBD-VVCA and mCherry-actin. Arrowheads point to the growing tip of a MBD-VVCA-labeled microtubule instantly recruiting actin. Time is in seconds; bar, 2 µm. (**B, C**) Representative frames taken from FRAP movies of cells transfected either with EGFP-tagged MBD-VVCA (B) or mCherry-MBD-VVCA (false-colored green in [C]) and EGFP-actin (false-colored red in [C] for clarity). Simultaneous imaging of MBD-VVCA was used to ensure bleaching of actin structures co-localizing with MBD-labeled microtubules. Circles in (B, C) indicate areas of bleaching and dashed lines enclose those regions used for measurements of fluorescence intensity changes over time. Time is in seconds; bars, 2 µm. (**D, E**) Fluorescence recovery curves obtained for EGFP-MBD-VVCA (D) or EGFP-actin (E). Data are means and standard errors of means (error bars) measured for each time point and construct as indicated. n values correspond to number of movies analyzed. Half times of fluorescence recovery (*t*_1/2_) were calculated from best exponential fits (green curves).

Although the induction of actin filament assembly on microtubules on average reduced microtubule dynamics as compared to cells expressing MBD alone (compare e.g. Movie S1 and Movie S3), it was still possible to explore actin dynamics during growth or shrinkage of individual microtubules. Interestingly, actin nucleation on growing microtubules did not lag behind the MBD-VVCA-binding to their growing tips, neither for the frame rate shown here (0.1 Hertz) (Figure 4A; Movie S7) nor at higher image acquisition frequencies (1 Hertz, not shown). We assume that the moderate average growth rates of microtubules of roughly 2.2 µm/min in these cells were not fast enough to detectably escape potent actin nucleation at the microtubule tip. These data indicate that MBD-VVCA binding can instantly drive actin filament nucleation at these sites. Furthermore, the shrinkage of microtubules again visualized with MBD-VVCA coincided with abrupt depolymerization or dissipation of actin filaments at microtubule tips (Figure S4; Movie S8), indicating that actin filaments on microtubules undergo rapid turnover, comparable to other Arp2/3 complex-dependent structures such as the lamellipodium [7]. To compare the dynamics of MBD-VVCA and actin at the surface of microtubules, we performed fluorescence recovery after photobleaching (FRAP) experiments (Figure 4B–E). Interestingly, MBD-VVCA bound along the length of microtubules continuously exchanged with the cytosolic pool with an average half time of 7.7 seconds (Figure 4B, D), similar to the turnover of the Arp2/3 complex activator WAVE at the lamellipodium tip [7]. This observation might explain the efficient activation of Arp2/3 complex on microtubules, since the residence times of a given Arp2/3 complex activator at a specific subcellular location could well be a limiting factor for activation efficiency. The turnover of actin filaments on microtubules was significantly slower than MBD-VVCA (*t_1/2_* = 16 sec, Figure 4C, E), again reminiscent of turnover rates observed for actin and Arp2/3 complex in the lamellipodium which are longer than the NPF at the tip, due to the treadmilling of the network [7]. These data indicate that the physical association of MBD-VVCA molecules with microtubules is sufficiently long to allow nucleation and elongation of individual actin filaments (see Figure 3). Turnover of the actin “network” is delayed however, because nucleation may not occur as instantly as MBD binding or because fluorescence in the network will have to recover by treadmilling or both. The turnover data of both MBD-VVCA and actin on microtubules best fitted bi-exponential models (Figure 4D, E), presumably due to the multitude of parameters potentially contributing to average exchange. More specifically, actin on microtubules may be lost by dissociation of entire filaments or oligomers and not just by depolymerization, and the residence times of individual MBD-VVCA molecules may vary depending on active engagement in Arp2/3 activation for instance or on steric restraints. Whatever the case, the data unequivocally show that the actin filament turnover observed in physiological, Arp2/3 complex-dependent structures such as the lamellipodium [8], [38] is largely recapitulated in this assay.

### Cortactin cannot activate Arp2/3 complex on microtubules

The experiments described above demonstrate the development of an assay that allows asking whether a given actin-binding protein can recruit proteins or protein machineries driving actin filament assembly. The type II NPF cortactin has been implicated in the regulation of multiple Arp2/3-dependent structures and co-localizes with Arp2/3 complex in lamellipodia and at sites of clathrin endocytosis [39], [40]. However, its precise functions *in viv*o are elusive, especially since genetic deletion in fibroblasts did not abolish lamellipodia formation or actin assembly accompanying endocytosis [38], [41]. Although these data indicated cortactin to be dispensable for Arp2/3 complex activation in these structures, they did not address directly whether cortactin is able in principle to activate Arp2/3 mediated actin assembly *in vivo*.

**Figure 5:**
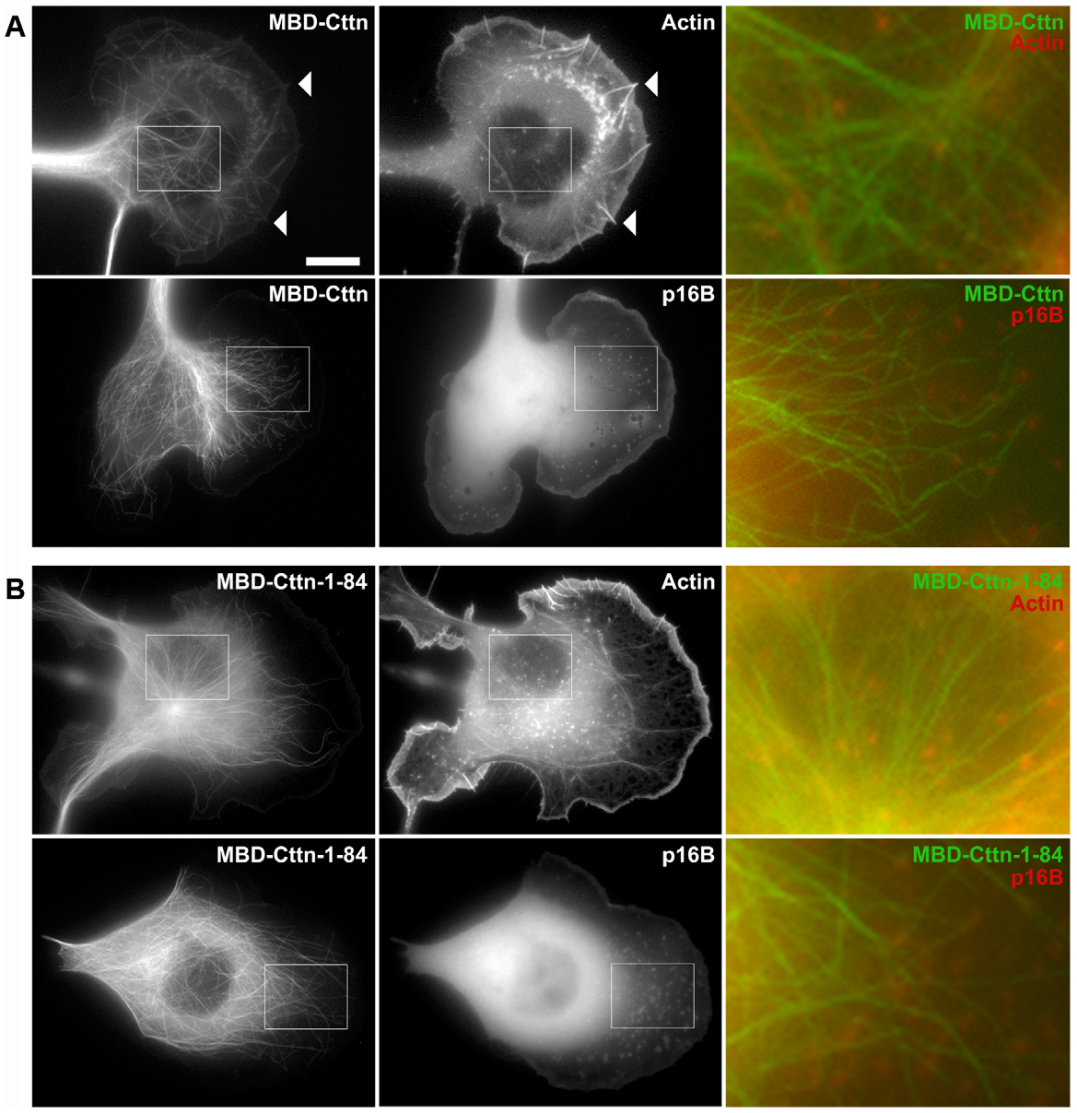
Cortactin does not drive actin assembly or Arp2/3 complex accumulation on microtubules. Epifluorescence images of live cells co-transfected with (**A**) EGFP-MBD-cortactin (MBD-Cttn) or (**B**) the EGFP-MBD-tagged N-terminus of Cortactin (MBD-Cttn 1–84) with mCherry-actin or mCherry-p16B as indicated. In neither case, a co-localization of the respective MBD-construct with actin or Arp2/3 complex was discernible. Merged images correspond to enlarged insets on the left. Arrowheads indicate co-localization of MBD-Cttn with microspikes. Bar, 10 µm.

To test this, full-length cortactin was fused to MBD in analogy to N-WASP-VVCA and co-expressed again with mCherry-tagged actin or p16B for visualization of Arp2/3 complex. Interestingly, the cortactin molecule was functional, since it mediated additional targeting of the EGFP-tagged fusion protein to its physiological localization in lamellipodia and microspikes (Figure 5A), and was expressed as a full-length protein as confirmed by western blotting (Figure S5). Although the MBD-cortactin chimera was mainly targeted to microtubules, this was not accompanied by accumulation of actin or Arp2/3 complex at these sites (Figure 5A; Movie S9 and Movie S10). Activation of Arp2/3 complex by cortactin *in vitro* requires interaction domains for both Arp2/3 complex and actin filaments [42], both of which were present in the chimera, however, the full-length molecule is potentially regulated by a complex network of additional interactions *in vivo* [43], which may influence the outcome of our assay. Thus, we also asked whether the isolated Arp2/3 binding surface of cortactin (N-terminal residues 1–84 [15], Figure 5B) might be able at least to recruit Arp2/3 complex. However, neither Arp2/3 complex nor actin (used as negative control) were targeted to microtubules in this case (Figure 5B; Movie S11 and Movie S12), indicating that binding of the N-terminus to Arp2/3 complex is not sufficient to ectopically target Arp2/3 complex *in vivo*. Based on these and our previous results obtained with genetic deletion of cortactin in fibroblasts [38], we conclude that the comparably weak Arp2/3 complex activation of cortactin observed *in vitro* does not suffice to potently activate Arp2/3 complex *in vivo*. The interaction of cortactin with Arp2/3 complex might indeed serve distinct functions, as suggested for instance by the observation of competitive binding with Arp2/3 complex between type I NPFs such as N-WASP and cortactin [44]. Finally, the intimate connection between cortactin and Arp2/3 complex function was underscored by the observation that full-length cortactin was co-recruited to microtubules with actin filaments induced by MBD-VVCA and Arp2/3 complex but not other nucleators (see also below), indicating that subcellular cortactin positioning *in vivo* occurs subsequently and not prior to Arp2/3 complex activation (Figure S6).

### Actin assembly on microtubules can be induced by distinct nucleation mechanisms

**Figure 6:**
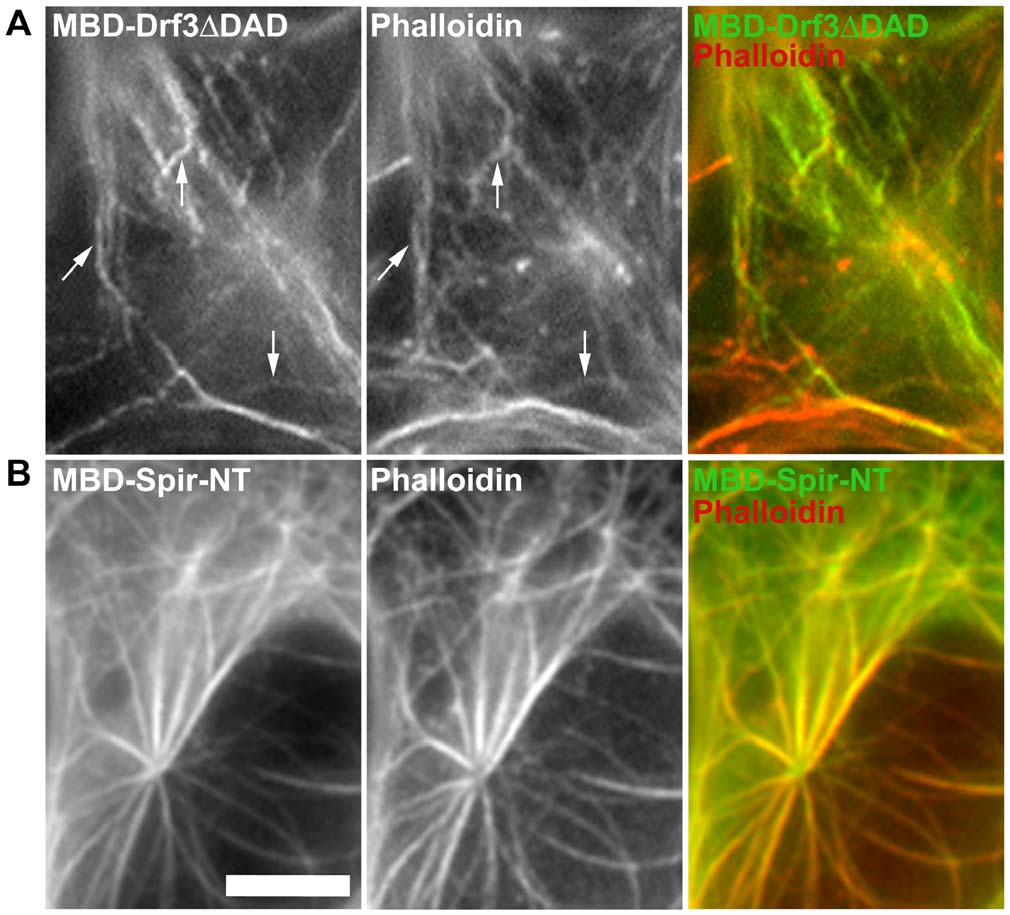
Actin nucleation on microtubules as induced by active Drf3/mDia2 and Spir. Phalloidin stainings of fixed B16-F1 cells expressing (**A**) MBD-tagged Drf3ΔDAD (mDia2-ΔDAD) and (**B**) MBD-Spir-NT. Merged images and arrows show co-localization of MBD-constructs with actin filaments. Bar, 5 µm.

As described above, our assay allows the induction of Arp2/3 complex-dependent actin assembly at ectopic sites *in vivo*. Next we asked whether actin polymerization could also be induced by Arp2/3 complex-independent nucleation mechanisms, e.g. by formins or Spir. Interestingly, fusion of the EGFP-MBD targeting unit with Drf3 lacking DAD (Drf3ΔDAD), an active variant of the formin Drf3 (also known as mDia2), which was previously concluded to potently stimulate filopodia formation *in vivo* through actin filament nucleation [45] was also capable of inducing actin assembly on microtubules (Figure 6A). Moreover, an N-terminal fragment of human Spir-1 (Spir-NT), which comprises all four WH2-domains, also mediated strong actin polymerization on microtubules (Figure 6B). Virtually identical results were obtained with the full-length variant of Spir-1 (data not shown). With both Drf3 and Spir-NT, actin assembly could be scored both in fixed cells stained with phalloidin to prove the presence of actin filaments (Figure 6A, B), and in live cells co-transfected with mCherry-actin (Figure S7A, B). The latter approach is more sensitive and easier to interpret. To confirm the specificity of each actin nucleation pathway, cells expressing EGFP-MBD-tagged Spir-NT or Drf3ΔDAD were counterstained with an antibody specific for the Arp2/3 complex subunit p16A (Figure S8A–C). Although the antibody strongly labeled lamellipodia and ruffles as well as vesicular structures in non-transfected cells as expected (Figure S8A), no co-localization was observed with ectopic Spir- or formin-induced actin filaments on microtubules (Figure S8B, C), indicating Arp2/3 independent actin assembly.

Collectively, these data demonstrate the general applicability of the microtubule platform to assay actin nucleation induced by different mechanisms *in vivo*.

### Employing the assay: probing the minimal requirements of Arp2/3 dependent actin assembly in vivo

**Figure 7:**
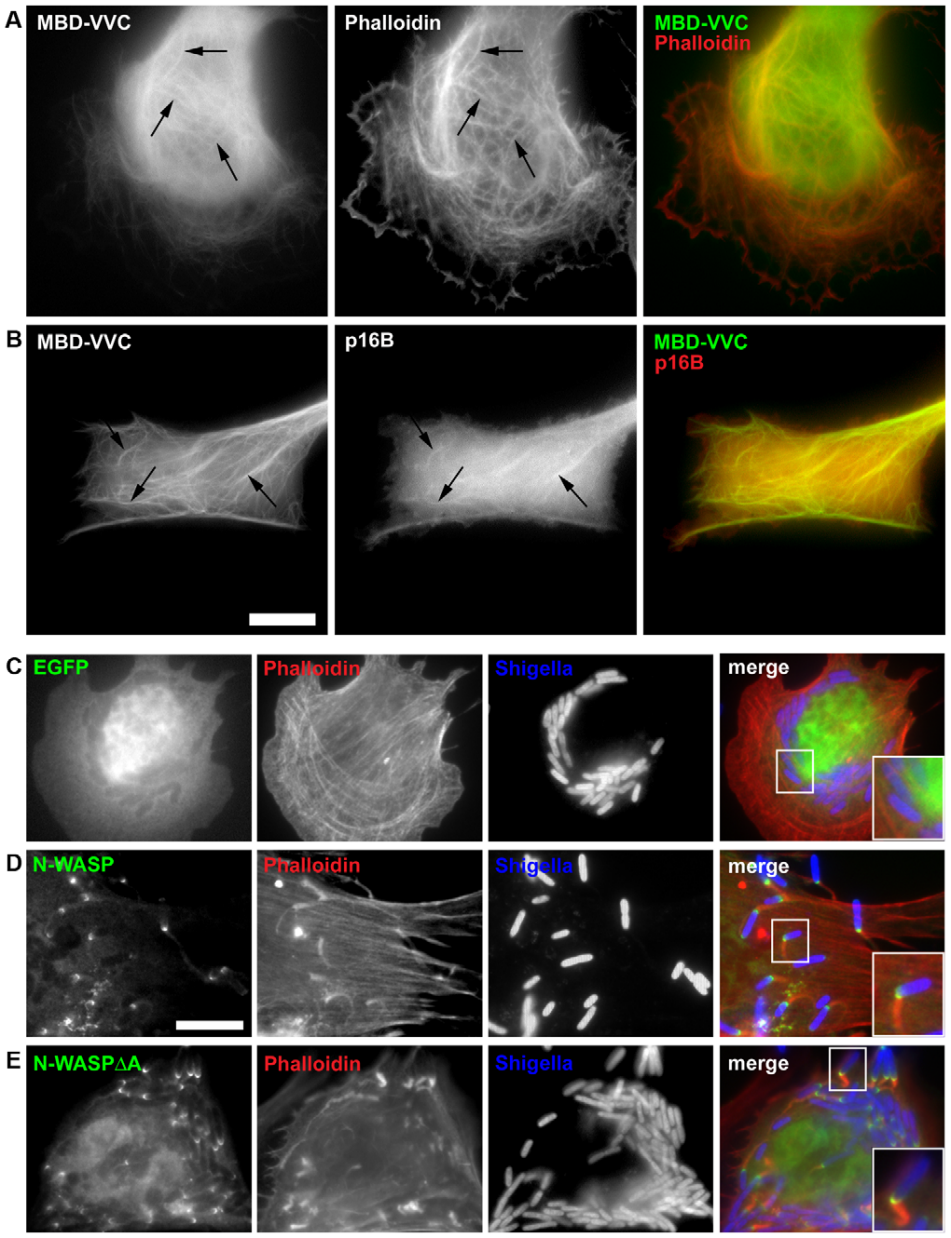
The acidic domain of N-WASP is dispensable for recruitment and activation of Arp2/3 complex. Epifluorescence images of cells transfected with EGFP-MBD-VVC and stained with phalloidin (**A**) or co-expressing fluorescently labeled p16B (**B**). Merged images and arrows indicate co-localization of MBD-VVC with actin filaments (A) and Arp2/3 complex (B). Bar, 10 µm. (**C–E**) Epifluorescence images of N-WASP-deficient cells transfected with EGFP (C), EGFP-N-WASP full length (D) or EGFP-N-WASPΔA (E) and infected with *S. flexneri* (*Shigella*). No actin tails are visible in cells transfected with EGFP alone (C), as expected [60], whereas both ectopically expressed N-WASP full length and N-WASPΔA bind to the surfaces of *Shigella*, and induce actin tail formation in N-WASP–deficient cells (see insets). Bar, 10 µm.

There is consensus that the WH2-domains (VV) in N-WASP-VVCA bind actin monomers and the acidic domain Arp2/3 complex, whereas the connector can interact with both [46]. All these interactions are considered essential for Arp2/3 dependent actin assembly [11]. The A-region harbors a tryptophan residue, the mutation of which to serine was sufficient to eliminate detectable Arp2/3 complex binding of WASP-VCA [47]. Notably, the same domain harboring the WH2 and connector regions had previously been observed to induce weak actin nucleation [48]. However, this was subsequently interpreted as an artifact of GST-induced dimerization [46]. A more recent study described strongly reduced but not abolished affinity of VVC for Arp2/3 complex, and actin assembly induced by this domain on synthetic vesicles *in vitro* [49]. Whether the acidic region might also be dispensable for Arp2/3 dependent actin assembly *in vivo* has remained unclear. We employed the MBD-assay to compare effects of microtubule targeting of VVCA (wildtype) with VVC and VV. For expression of all variants see Figure S9. MBD-VV failed to induce actin and Arp2/3 complex recruitment (Figure S2, see above). However, expression of MBD-VVC induced significant actin and Arp2/3 complex accumulation on microtubules (Figure 7A, B), although less robustly especially in case of Arp2/3 complex than usually observed for VVCA. This conclusion is based on the fact that accumulation on microtubules was more difficult to distinguish from the cytosolic fraction than observed in VVCA-expressors (compare Figure 1 and Figure 7). Furthermore, when counting the number of cells capable in principle of actin accumulation for each construct, it became evident that the frequency of actin co-localization with microtubules (97.2% for VVCA-expressors [n = 252]) was significantly reduced to 43.2% (n = 264) in case of VVC (p<0.001), whereas no single co-localization was scored in case of VV (n = 278) (Figure S10). The accumulation of Arp2/3 complex followed a similar trend, although the detection frequency on microtubules was generally reduced for all constructs (Figure S10). Nevertheless, Arp2/3 complex enrichment could clearly be detected also with VVC (10.7%; n = 190), albeit much less robustly than with VVCA (69.4%; n = 193). Again, no recruitment could be scored with VV alone (n = 271) (Figure S10). These data strongly suggest that the acidic domain contributes to, but is not essential for Arp2/3 complex-dependent actin assembly *in vivo*. The physiologic relevance of this observation was confirmed by comparing N-WASP-dependent actin tail formation induced by intracellular *Shigella* in N-WASP null cells reconstituted either with EGFP-tagged full length N-WASP or N-WASP lacking the acidic domain (N-WASPΔA) (Figure 7C–E). Interestingly, both N-WASP variants were capable of driving actin tail formation, although reduced efficiency of N-WASPΔA was reflected e.g. by increased expression levels required for inducing prominent actin tails (not shown). These results demonstrate for the first time the dispensability of the acidic domain in an N-WASP-dependent process *in vivo*, and call for revision of our views on the functional relevance of this domain in both type I and II NPFs.

## Discussion

The nucleation of actin filaments is key to numerous processes regulating cell division, morphogenesis, migration, signaling and host-pathogen interaction. A constantly increasing number of molecules can influence actin nucleation activity *in vitro*, but how and where exactly they function *in vivo* is often unknown. Whether or not actin assembly mediated by a given factor *in vitro* translates into *bona fide* actin nucleation activity in the cytoplasm frequently remains unclear. Here we introduce a novel and robust assay to analyze actin nucleation ectopically targeted to the surface of microtubules. Ectopic actin assembly had previously been induced on mitochondria [50], [51], [52] or late endosomes in *Dictyostelium* [53]. The use of endosomes is complicated by the fact that these structures do assemble actin filaments by themselves to counteract their fusion [54]. As opposed to this, it is quite safe to assume that a construct engineered to induce actin assembly on the surface of microtubules will find essentially no endogenous actin and thus other actin binding proteins that might aid the ectopic actin assembly process. The conclusion that Arp2/3 complex activation in live cells is feasible in the absence of the acidic domain of the N-WASP C-terminus would not have been legitimate on subcellular structures suspected to recruit endogenous actin. Mitochondria recruit little actin [55], but are highly dynamic structures undergoing fusion and fission events within seconds [56], thus compromising the in-depth analysis of actin assembly by live-cell imaging. This is most critical for turnover studies such as those using FRAP, since the relatively slow turnover rates observed for actin and actin regulators can only reliably be determined on comparably rigid, and thus stable structures. Likewise, the topology and comparably firm surface of microtubules will both be advantageous for successful relocation and for detailed analyses of the ultrastructure of actin networks generated by distinct actin nucleators.

Actin polymerization could be induced irrespective of the molecular mechanism of nucleation, demonstrating the versatility of the assay. In principle this approach is applicable to any transfectable cell line, irrespective of model organism or cell type. In our hands, transfection with MBD-VVCA of murine NIH 3T3 or fish CAR fibroblasts also caused robust actin assembly on microtubules (not shown). However, we recommend analyses best be completed by 24–48 hours after transfection. It should be pointed out that the strongly-induced reorganization of the actin cytoskeleton might affect additional, actin-dependent processes, so analyses of MBD-construct expressors should be restricted to actin assembly events on the surface of microtubules. Nevertheless, spindle formation and cytokinesis could still be observed in cells expressing MBD-VVCA (not shown), indicating that these processes are not generally blocked, but the treatment could interfere with efficiency or frequency of their occurrence. Moreover, MBD-VVCA-induced reprograming of Arp2/3 complex activation onto the microtubule surface abolished lamellipodia formation, which is not surprising given that Arp2/3 complex is considered to be essential for the formation of these protrusions [57] and that simple sequestration of Arp2/3 complex in the cytosol also interfered with lamellipodia formation [58]. In contrast, cells expressing MBD-Spir-NT appeared more likely to form lamellipodia (not shown), consistent with the absence of a reported function for Spir in the formation of these structures [24].

The assay is ideally suited for the analysis of co-recruitment of additional regulators (exemplified here by cortactin), and can be extended to tuning output actin assembly by RNAi-mediated suppression of specific components. Furthermore, assaying actin assembly induced on the same structure by distinct nucleators upon knockdown or knockout of a given factor will allow to directly uncover its potential differential functions in distinct actin assembly processes.

Combination of the simple actin accumulation readout with high content screening should facilitate identification of novel activators or inhibitors of specific actin nucleation mechanisms *in vivo*. This way the assay will help to increase our understanding of the complex interplay of different actin assembly mechanisms in normal and diseased cells.

## Methods

### DNA constructs

EGFP tagged human β-actin was purchased from Clontech (Mountain View, CA, USA), and the following constructs were described previously: mCherry-actin [59], EGFP-N-WASP and pEGFP-C3-VVCA [60]. EBFP2-actin was obtained by fusing human β-actin into pEBFP2-C1, kindly provided by Dr. Robert E. Campbell [61]. mCherry-p16B was obtained by exchanging EGFP in EGFP-p16B [31] for mCherry. For generation of microtubule targeting constructs, the MBD encoded by residues 688–1151 of human MAP4, (isoform 3; genebank accession: U19727) was amplified with pMEP-MAP4 as template [62], kindly provided by Dr. Martin Gullberg (Umeå, Sweden) and using primers 5′-gacatgtacaccccaccgaac-3′ (forward) and 5′-gtcatctgtacatgcttgtctcc-3′ (reverse), thereby introducing BsrGI sites. For the control vector, the MBD-fragment was fused into pEGFP-C1 (Clontech) using BsrGI digestion. For EGFP-MBD-VVCA, the fragment was fused in frame into pEGFP-C3-VVCA [60]. Fusion with other cDNAs of interest was routinely done in an analogous fashion based on constructs harboring the respective N-terminally EGFP-tagged sequences. These included EGFP-Drf3ΔDAD [45], EGFP-fascin [63] or EGFP-cortactin. For generation of the latter, murine cortactin (genebank accession: NM_007803) was cloned into pEGFP-C1 vector. mCherry-cortactin, mCherry-VVCA and mCherry-Spir-NT were generated by exchanging EGFP in respective vectors described above for mCherry.

MBD-Spir-NT was generated by fusing Spir-NT (see below) into pEGFP-MBD-C1. Spir-NT (the N-terminal KIND and four WH2 domains) corresponded to residues 2–402 of human Spir-1 (isoform 2). Cttn 1–84 was made by amplification of the N-terminal 84 amino acids of murine cortactin using primers 5′-gagagaattcatgtggaaagcctctgc-3′ (forward) and 5′-gagagtcgacatagccgtgggaagcctt-3′ (reverse) and cloning into EGFP-MBD-C2. The sequence encoding the actin-binding domain of α-actinin comprising both calponin homology domains [64] was amplified from EGFP-α-actinin [65], using the following primers: 5′-gagagaattcatggaccattatgattctc-3′ (forward) and 5′-gagagtcgactgctgtctccgccttctgg-3′ (reverse). The PCR fragment was fused into EGFP-MBD-C2 or EGFP-C2 as control. MBD-VV (amino acids 392–460 of murine N-WASP) was amplified from EGFP-VVCA [60] using primers 5′-gagagaattccatcaagttccagctcct-3′ (forward) and 5′-gagagtcgacagtgggtgcgggtgttgg-3′ (reverse), and MBD-VVC (amino acids 392–483 of murine N-WASP) using primers 5′-gagagaattccatcaagttccagc-3′ (forward) and 5′-tgtcgtcgacttattcatctgagga-3′ (reverse). Both fragments were ligated into EGFP-MBD-C2. To obtain N-WASPΔA, amino acid residues 1–483 of murine N-WASP [60] were amplified with primers 5′-gagagaattcatgagctcgggccagcag-3′ (forward) and 5′-gagagtcgacttattcatctgaggaatga-3′ (reverse) and were cloned into EGFP-C2. All PCR fragments were sequenced to ensure correct amplification.

### Cells, transfections and western blotting

Mouse melanoma cells (B16-F1) were purchased from American Type Culture Collection (ATCC CRL-6323) and were cultured in DMEM (Invitrogen, Germany) with 10% FCS (PAA Laboratories, Austria) and 2 mM glutamine (Invitrogen) at 37°C in the presence of 7.5% CO2. Cells were transfected using Superfect (Quiagen) according to manufacturer's instructions. One day after transfection, B16-F1 cells were seeded onto coverslips coated with 25 µg/ml laminin (Sigma-Aldrich) and either examined by video microscopy or fixed and processed for immunolabeling or electron microscopy. Ectopic expression of EGFP-tagged proteins was analyzed by western blotting using standard protocols. Monoclonal anti-GFP antibody (clone 101G4) is available from Synaptic Systems (Göttingen, Germany). N-WASP-deficient cells [60] were maintained and transfected as described.

### *Shigella* actin tail formation

Infections with *S. flexneri* were performed as described [60]. Briefly, N-WASP-deficient cells were seeded onto fibronectin-coated coverslips, transfected and infected with *Shigella* (M90T wild-type invasive strain serotype 5) two days after plating. Bacteria were slowly centrifuged to bring them in close proximity to the host cell layer, and allowed to infect for 1.5 h before extracellular bacteria were killed using 50 µg/ml gentamicin (Sigma). Cells were fixed for 20 min with 4% paraformaldehyde (PFA) and 0.1%Triton X100 in phosphate-buffered saline (PBS), and subjected to immunolabeling.

### Light and video microscopy

Phalloidin stainings and immunolabeling experiments were performed as described [66]. Transfected cells were seeded onto glass coverslips coated with laminin (25 µg/ml) or fibronectin (25 µg/ml, for N-WASP^−/−^ cells), and fixed with 4% paraformaldehyde (PFA) in PBS (37°C) for 20 min followed by extraction with 0.1% Triton X100 for 1 min. Subsequently, samples were stained with Alexa594- or Alexa350-labelled phalloidin to detect actin filaments, anti-α-tubulin (clone 3A2, Synaptic Systems), monoclonal anti-p16A antibody (clone 323H3 [30]) for staining of the Arp2/3 complex or polyclonal anti-*Shigella* antibody (Abcam), followed by secondary, Alexa594-labelled goat anti mouse antibody or Alexa350-labelled goat anti rabbit antibody (both Invitrogen).

Light microscopy was performed on an inverted microscope (Axiovert 100TV; Carl Zeiss, Jena, Germany) using standard epifluorescence illumination (light source HXP120, Zeiss) and 63×/NA1.4 or 100×/NA1.4 plan-apochromatic objectives. Images were acquired with a back-illuminated, cooled charge-coupled-device camera (CoolSNAP HQ2, Photometrics, Tucson, AZ, USA) driven by Metamorph software (Molecular Devices Corp., Downingtown, PA, USA).

For video microscopy and FRAP experiments, cells were mounted in an open, heated chamber (Warner Instruments, Reading, United Kingdom) at 37°C. Actin assembly and Arp2/3 complex accumulation induced by VVCA, VVC *versus* VV were quantified using live cell imaging, scoring cells as detailed in the legend to Figure S10. Statistical analyses were done using OriginPro 8.5 (OriginLab Corporation, Northampton, USA). FRAP experiments were performed with minor modifications as described before [7] using a double-scan-headed confocal microscope (Fluoview1000, Olympus, Hamburg, Germany) equipped with a 100×/1.45 PlanApo TIRF objective (Olympus). Circular regions drawn around individual microtubules were bleached with a 405 nm diode using “tornado mode”. Movies were acquired at a scanning rate of 1.644 s per frame. Average fluorescence intensities of microtubules or background were measured with Metamorph software (Molecular Devices Corp.) before and after bleaching. The fluorescence intensity of the last frame before bleaching was defined as maximum and normalized to 1. Background fluorescence was subtracted and data were analyzed using SigmaPlot 11.0 and Microsoft Excel 2000. Exponential curves in Figure 4 corresponded to best fits of means. Fitted data followed equation y = a(1−exp(−bx))+c(1−exp(−dx)), with a = 0.5628, b = 0.1583, c = 0.3708 and d = 0.0253 for MBD-VVCA and a = 0.2743, b = 0.3601, c = 0.6246 and d = 0.0164 for actin. Half times of recovery (*t_1/2_*) were calculated by solving the corresponding equations at 50% of the maximal recovery value derived from each fitted curve.

### Electron tomography

Correlated live cell imaging and electron tomography was performed essentially as described in [67]. Briefly, B16-F1 melanoma cells were co-transfected with EGFP-MBD-VVCA and mCherry-actin and plated onto formvar-coated coverslips. Cells expressing both constructs were located by fluorescence microscopy and imaged before and after fixation in a mixture of 0.5% Triton X100 and 0.25% glutaraldehyde in cytoskeleton buffer (10 mM MES, 150 mMNaCl, 5 mM EGTA, 5 mM glucose and 5 mM MgCl_2_, at pH 6.1). After an initial fixation of 1 min the cells were post-fixed in 2% glutaraldehyde containing 10 µg/ml phalloidin and stored in the same mixture at 4°C. The film was subsequently peeled from the coverslip, an EM grid positioned over the area containing the cell of interest and the grid negatively-stained with 6% sodium silicotungstate containing 10 µg/ml phalloidin and a 10 nm gold sol. The cell was relocated in the electron microscope (FEI Tecnai F30 Polara operating at 300 kV) and tomographic series recorded around two orthogonal axes. Re-projections from the tilt series were generated using IMOD software from the Boulder Laboratory for 3D Electron Microscopy of Cells, using the gold particles as fiducials for alignment [68].

## Supporting Information

Figure S1 **MBD or VVCA alone do not target actin to microtubules.** Epifluorescence images of cells co-expressing (**A**) EGFP-tagged MBD-VVCA and mCherry-actin as control or (**B**) EGFP-tagged MBD and mCherry-actin or mCherry-p16B as indicated. Note that MBD-VVCA and actin co-localize, whereas no overlap of MBD with actin or MBD with p16B is visible. Bar, 10 µm. (**C**) Phalloidin staining (blue in merge) and immunolabeling with anti-α-tubulin antibodies (red in merge) of a cell transfected with EGFP-VVCA (green in merge). Merge corresponds to boxed regions in left panels. Since VVCA does not target to microtubules, they are completely devoid of actin filaments. Bar, 10 µm. (**D**) Immunoblot confirming expression of EGFP-tagged MBD and MBD-VVCA at appropriate molecular weights.
(TIF)

Figure S2 **The WH2-domains of N-WASP cannot nucleate actin filaments on microtubules.** Selected frames from time-lapse movie of B16-F1 cell co-expressing EGFP-tagged MBD-VV and mCherry-actin (upper panel) or mCherry-p16B (lower panel). Insets on the left are magnified in merged images on the right, revealing the absence of co-localization of MBD-VV (green in merge) with actin or Arp2/3 complex (red in merges). Bar, 10 µm.
(TIF)

Figure S3 **Subcellular localization of the actin-binding domain of α-actinin (ABD) and its expression compared to MBD-tagged ABD and fascin.** (**A**) Phalloidin staining of a B16-F1 cell ectopically expressing EGFP-ABD revealing that the ABD of α-actinin robustly associates with actin networks and bundles located in the lamellipodium and the lamella behind, as expected. Bar, 5 µm. (**B**) Verification of correct expression of EGFP-tagged MBD-fascin, ABD and MBD-ABD as indicated.
(TIF)

Figure S4 **MBD-VVCA and polymerized actin dissociate instantly from shrinking microtubules.** Selected frames from time-lapse movie of B16-F1 cell co-expressing EGFP-tagged MBD-VVCA and mCherry-actin as indicated. Arrowheads point to a shrinking microtubule. Time is in seconds; bar, 2 µm.
(TIF)

Figure S5 **Expression control of MBD-Cttn and MBD-Cttn 1–84.** Expression of EGFP-tagged MBD-Cortactin (MBD-Cttn) and its N-terminal 84 amino acids (MBD-Cttn 1–84) as verified by immunoblotting.
(TIF)

Figure S6 **Cortactin is recruited to Arp2/3 but not Spir-NT-induced actin assemblies.** Epifluorescence images of live cells co-transfected with (**A**) mCherry-MBD-VVCA and EGFP-cortactin (false-colored in merge for clarity) or (**B**) EGFP-Spir-NT and mCherry-cortactin. Merged image and arrows in (A) show significant accumulation of cortactin at MBD-VVCA-stimulated actin structures. In contrast, no targeting to microtubules decorated with Spire-NT was discernible. Bar, 5 µm.
(TIF)

Figure S7 **Actin polymerization on microtubules by MBD-Drf3ΔDAD and MBD-Spir-NT.** Live cell imaging of B16-F1 cells co-expressing mCherry-actin and (**A**) EGFP-tagged MBD-Drf3ΔDAD or (**B**) MBD-Spir-NT. Merged images and arrows indicate robust co-localization of respective MBD-construct with actin. Bar, 5 µm.
(TIF)

Figure S8 **MBD-Drf3ΔDAD and MBD-Spir-NT nucleate actin filaments on microtubules independently of Arp2/3 complex.** Immunolabeling experiments showing Arp2/3 complex localization (p16A) in (**A**) non-transfected control cell or in cells expressing EGFP-tagged (**B**) MBD-Drf3ΔDAD or (**C**) MBD-Spir-NT. Bar, 5 µm.
(TIF)

Figure S9 **Immunoblot showing expression of EGFP-tagged MBD-VV and MBD-VVC compared to MBD and MBD-VVCA.** Asterisk marks an additional, truncated product detected by anti-GFP antibodies (MBD-VVC lane) with an approximate size of 50 kDa, thus unable presumably to interfere with microtubule targeting and thus actin assembly induced by the full length fragment (Figure 1A).
(TIF)

Figure S10 **MBD-VVCA co-localizes more frequently with actin and p16B compared to MBD-VVC and MBD-VV.** Cells were transfected with MBD-VVCA, MBD-VVC or MBD-VV and additionally with either actin or p16B. Living cells were classified into categories: actin (**A**) or p16B (**B**) co-localizing or not co-localizing with the respective MBD-construct on microtubules, as indicated. Cells in which actin or p16B accumulation on microtubules could not be determined due to overexpression of either construct were classified “ambiguous”. Data are means and standard errors of means (error bars) and n values correspond to number of cells analyzed. The differences between MBD-VVCA- and MBD-VVC-expressors co-localizing with actin or p16B were confirmed to be statistically significant by two-sided two-sample *t* test.
(TIF)

Movie S1 **MBD-VVCA induces actin assembly on the surface of microtubules.**
(MOV)

Movie S2 **MBD-VVCA-induced actin assembly on microtubules is accompanied by Arp2/3 complex recruitment.**
(MOV)

Movie S3 **MBD alone fails to attract actin assembly to microtubules.**
(MOV)

Movie S4 **Arp2/3 complex is not targeted to microtubules associated with MBD alone.**
(MOV)

Movie S5 **MBD-VV cannot stimulate actin assembly on microtubules.**
(MOV)

Movie S6 **MBD-VV cannot recruit Arp2/3 complex to microtubules.**
(MOV)

Movie S7 **Actin is instantly assembled at the tips of growing microtubules.**
(MOV)

Movie S8 **Actin is abruptly disassembled at the tips of shrinking microtubules.**
(MOV)

Movie S9 **MBD-cortactin cannot stimulate actin assembly on microtubules.**
(MOV)

Movie S10 **MBD-cortactin cannot recruit Arp2/3 complex to microtubules.**
(MOV)

Movie S11 **MBD-tagged, N-terminal cortactin cannot target actin to microtubules.**
(MOV)

Movie S12 **MBD-tagged, N-terminal cortactin cannot target Arp2/3 complex to microtubules.**
(MOV)

## References

[1] Albiges-Rizo C, Destaing O, Fourcade B, Planus E, Block MR (2009). Actin machinery and mechanosensitivity in invadopodia, podosomes and focal adhesions.. J Cell Sci.

[2] Insall RH, Machesky LM (2009). Actin dynamics at the leading edge: from simple machinery to complex networks.. Dev Cell.

[3] Cingolani LA, Goda Y (2008). Actin in action: the interplay between the actin cytoskeleton and synaptic efficacy.. Nat Rev Neurosci.

[4] Dustin ML (2007). Cell adhesion molecules and actin cytoskeleton at immune synapses and kinapses.. Curr Opin Cell Biol.

[5] Pantaloni D, Le Clainche C, Carlier MF (2001). Mechanism of actin-based motility.. Science.

[6] Wegner A (1976). Head to tail polymerization of actin.. Journal of molecular biology.

[7] Lai FP, Szczodrak M, Block J, Faix J, Breitsprecher D (2008). Arp2/3 complex interactions and actin network turnover in lamellipodia.. EMBO J.

[8] Wang YL (1985). Exchange of actin subunits at the leading edge of living fibroblasts: possible role of treadmilling.. J Cell Biol.

[9] Chesarone MA, Goode BL (2009). Actin nucleation and elongation factors: mechanisms and interplay.. Curr Opin Cell Biol.

[10] Goley ED, Welch MD (2006). The ARP2/3 complex: an actin nucleator comes of age.. Nature reviews Molecular cell biology.

[11] Campellone KG, Welch MD (2010). A nucleator arms race: cellular control of actin assembly.. Nat Rev Mol Cell Biol.

[12] Rottner K, Hanisch J, Campellone KG (2010). WASH, WHAMM and JMY: regulation of Arp2/3 complex and beyond.. Trends in cell biology.

[13] Stradal TE, Scita G (2006). Protein complexes regulating Arp2/3-mediated actin assembly.. Current opinion in cell biology.

[14] Stradal TE, Rottner K, Disanza A, Confalonieri S, Innocenti M (2004). Regulation of actin dynamics by WASP and WAVE family proteins.. Trends in cell biology.

[15] Ammer AG, Weed SA (2008). Cortactin branches out: roles in regulating protrusive actin dynamics.. Cell Motil Cytoskeleton.

[16] Chesarone MA, DuPage AG, Goode BL (2010). Unleashing formins to remodel the actin and microtubule cytoskeletons.. Nature reviews Molecular cell biology.

[17] Schonichen A, Geyer M (2010). Fifteen formins for an actin filament: a molecular view on the regulation of human formins.. Biochimica et biophysica acta.

[18] Quinlan ME, Heuser JE, Kerkhoff E, Mullins RD (2005). Drosophila Spire is an actin nucleation factor.. Nature.

[19] Ahuja R, Pinyol R, Reichenbach N, Custer L, Klingensmith J (2007). Cordon-bleu is an actin nucleation factor and controls neuronal morphology.. Cell.

[20] Chereau D, Boczkowska M, Skwarek-Maruszewska A, Fujiwara I, Hayes DB (2008). Leiomodin is an actin filament nucleator in muscle cells.. Science.

[21] Okada K, Bartolini F, Deaconescu AM, Moseley JB, Dogic Z (2010). Adenomatous polyposis coli protein nucleates actin assembly and synergizes with the formin mDia1.. The Journal of cell biology.

[22] Takano K, Watanabe-Takano H, Suetsugu S, Kurita S, Tsujita K (2010). Nebulin and N-WASP cooperate to cause IGF-1-induced sarcomeric actin filament formation.. Science.

[23] Bosch M, Le KH, Bugyi B, Correia JJ, Renault L (2007). Analysis of the function of Spire in actin assembly and its synergy with formin and profilin.. Molecular cell.

[24] Kerkhoff E (2010). Actin dynamics at intracellular membranes: The Spir/formin nucleator complex.. European journal of cell biology.

[25] Quinlan ME, Hilgert S, Bedrossian A, Mullins RD, Kerkhoff E (2007). Regulatory interactions between two actin nucleators, Spire and Cappuccino.. The Journal of cell biology.

[26] Renault L, Bugyi B, Carlier MF (2008). Spire and Cordon-bleu: multifunctional regulators of actin dynamics.. Trends in cell biology.

[27] Small JV, Stradal T, Vignal E, Rottner K (2002). The lamellipodium: where motility begins.. Trends Cell Biol.

[28] Rohatgi R, Ma L, Miki H, Lopez M, Kirchhausen T (1999). The interaction between N-WASP and the Arp2/3 complex links Cdc42-dependent signals to actin assembly.. Cell.

[29] Aizawa H, Emori Y, Mori A, Murofushi H, Sakai H (1991). Functional analyses of the domain structure of microtubule-associated protein-4 (MAP-U).. J Biol Chem.

[30] Millard TH, Behrendt B, Launay S, Futterer K, Machesky LM (2003). Identification and characterisation of a novel human isoform of Arp2/3 complex subunit p16-ARC/ARPC5.. Cell Motil Cytoskeleton.

[31] Rottner K, Kaverina IN, Stradal TEB, Celis JE (2006). Cytoskeleton Proteins.. Cell Biology, A Laboratory Handbook. 3 ed.

[32] Okreglak V, Drubin DG (2010). Loss of Aip1 reveals a role in maintaining the actin monomer pool and an in vivo oligomer assembly pathway.. J Cell Biol.

[33] Co C, Wong DT, Gierke S, Chang V, Taunton J (2007). Mechanism of actin network attachment to moving membranes: barbed end capture by N-WASP WH2 domains.. Cell.

[34] Adams JC (2004). Roles of fascin in cell adhesion and motility.. Current opinion in cell biology.

[35] Vignjevic D, Kojima S, Aratyn Y, Danciu O, Svitkina T (2006). Role of fascin in filopodial protrusion.. J Cell Biol.

[36] Sjoblom B, Salmazo A, Djinovic-Carugo K (2008). Alpha-actinin structure and regulation.. Cellular and molecular life sciences: CMLS.

[37] Nemethova M, Auinger S, Small JV (2008). Building the actin cytoskeleton: filopodia contribute to the construction of contractile bundles in the lamella.. J Cell Biol.

[38] Lai FP, Szczodrak M, Oelkers JM, Ladwein M, Acconcia F (2009). Cortactin promotes migration and platelet-derived growth factor-induced actin reorganization by signaling to Rho-GTPases.. Mol Biol Cell.

[39] Cao H, Orth JD, Chen J, Weller SG, Heuser JE (2003). Cortactin is a component of clathrin-coated pits and participates in receptor-mediated endocytosis.. Mol Cell Biol.

[40] Kaksonen M, Peng HB, Rauvala H (2000). Association of cortactin with dynamic actin in lamellipodia and on endosomal vesicles.. J Cell Sci.

[41] Tanaka S, Kunii M, Harada A, Okabe S (2009). Generation of cortactin floxed mice and cellular analysis of motility in fibroblasts.. Genesis.

[42] Uruno T, Liu J, Zhang P, Fan Y, Egile C (2001). Activation of Arp2/3 complex-mediated actin polymerization by cortactin.. Nat Cell Biol.

[43] Tehrani S, Tomasevic N, Weed S, Sakowicz R, Cooper JA (2007). Src phosphorylation of cortactin enhances actin assembly.. Proc Natl Acad Sci U S A.

[44] Padrick SB, Cheng HC, Ismail AM, Panchal SC, Doolittle LK (2008). Hierarchical regulation of WASP/WAVE proteins.. Mol Cell.

[45] Block J, Stradal TE, Hanisch J, Geffers R, Kostler SA (2008). Filopodia formation induced by active mDia2/Drf3.. J Microsc.

[46] Kelly AE, Kranitz H, Dotsch V, Mullins RD (2006). Actin binding to the central domain of WASP/Scar proteins plays a critical role in the activation of the Arp2/3 complex.. J Biol Chem.

[47] Marchand JB, Kaiser DA, Pollard TD, Higgs HN (2001). Interaction of WASP/Scar proteins with actin and vertebrate Arp2/3 complex.. Nat Cell Biol.

[48] Hufner K, Higgs HN, Pollard TD, Jacobi C, Aepfelbacher M (2001). The verprolin-like central (vc) region of Wiskott-Aldrich syndrome protein induces Arp2/3 complex-dependent actin nucleation.. J Biol Chem.

[49] Delatour V, Helfer E, Didry D, Le KH, Gaucher JF (2008). Arp2/3 controls the motile behavior of N-WASP-functionalized GUVs and modulates N-WASP surface distribution by mediating transient links with actin filaments.. Biophys J.

[50] Kessels MM, Qualmann B (2002). Syndapins integrate N-WASP in receptor-mediated endocytosis.. EMBO J.

[51] Pistor S, Chakraborty T, Niebuhr K, Domann E, Wehland J (1994). The ActA protein of Listeria monocytogenes acts as a nucleator inducing reorganization of the actin cytoskeleton.. EMBO J.

[52] Fradelizi J, Noireaux V, Plastino J, Menichi B, Louvard D (2001). ActA and human zyxin harbour Arp2/3-independent actin-polymerization activity.. Nature cell biology.

[53] Schmauch C, Claussner S, Zoltzer H, Maniak M (2009). Targeting the actin-binding protein VASP to late endosomes induces the formation of giant actin aggregates.. Eur J Cell Biol.

[54] Drengk A, Fritsch J, Schmauch C, Ruhling H, Maniak M (2003). A coat of filamentous actin prevents clustering of late-endosomal vacuoles in vivo.. Curr Biol.

[55] Zhang J, Fonovic M, Suyama K, Bogyo M, Scott MP (2009). Rab35 controls actin bundling by recruiting fascin as an effector protein.. Science.

[56] Chan DC (2006). Mitochondria: dynamic organelles in disease, aging, and development.. Cell.

[57] Steffen A, Faix J, Resch GP, Linkner J, Wehland J (2006). Filopodia formation in the absence of functional WAVE- and Arp2/3-complexes.. Molecular biology of the cell.

[58] Machesky LM, Insall RH (1998). Scar1 and the related Wiskott-Aldrich syndrome protein, WASP, regulate the actin cytoskeleton through the Arp2/3 complex.. Current biology: CB.

[59] Koestler SA, Auinger S, Vinzenz M, Rottner K, Small JV (2008). Differentially oriented populations of actin filaments generated in lamellipodia collaborate in pushing and pausing at the cell front.. Nat Cell Biol.

[60] Lommel S, Benesch S, Rottner K, Franz T, Wehland J (2001). Actin pedestal formation by enteropathogenic Escherichia coli and intracellular motility of Shigella flexneri are abolished in N-WASP-defective cells.. EMBO Rep.

[61] Ai HW, Shaner NC, Cheng Z, Tsien RY, Campbell RE (2007). Exploration of new chromophore structures leads to the identification of improved blue fluorescent proteins.. Biochemistry.

[62] Holmfeldt P, Brattsand G, Gullberg M (2002). MAP4 counteracts microtubule catastrophe promotion but not tubulin-sequestering activity in intact cells.. Curr Biol.

[63] Adams JC, Schwartz MA (2000). Stimulation of fascin spikes by thrombospondin-1 is mediated by the GTPases Rac and Cdc42.. J Cell Biol.

[64] Gimona M, Djinovic-Carugo K, Kranewitter WJ, Winder SJ (2002). Functional plasticity of CH domains.. FEBS Lett.

[65] Breitbach K, Rottner K, Klocke S, Rohde M, Jenzora A (2003). Actin-based motility of Burkholderia pseudomallei involves the Arp 2/3 complex, but not N-WASP and Ena/VASP proteins.. Cell Microbiol.

[66] Steffen A, Rottner K, Ehinger J, Innocenti M, Scita G (2004). Sra-1 and Nap1 link Rac to actin assembly driving lamellipodia formation.. EMBO J.

[67] Urban E, Jacob S, Nemethova M, Resch GP, Small JV (2010). Electron tomography reveals unbranched networks of actin filaments in lamellipodia.. Nat Cell Biol.

[68] Auinger S, Small JV (2008). Correlated light and electron microscopy of the cytoskeleton.. Methods Cell Biol.

