# Supplementary figures and images for: Microtubules as Platforms for Assaying Actin Polymerization In Vivo
Source: PLoS One. 2011 May 16;6(5):e19931. doi: 10.1371/journal.pone.0019931 (PMC3095617; doi:10.1371/journal.pone.0019931)

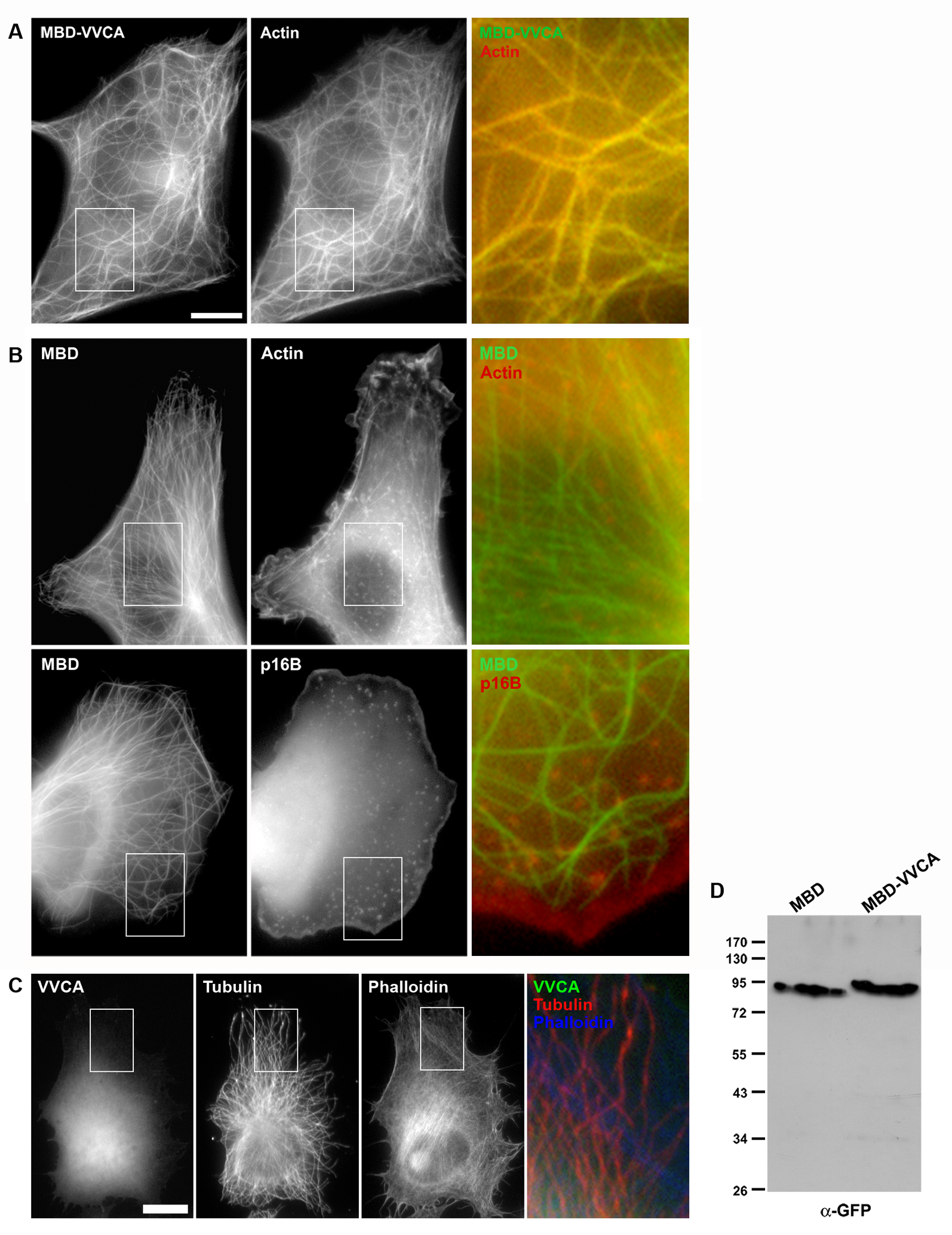

Supplement: Figure S1 — MBD or VVCA alone do not target actin to microtubules. Epifluorescence images of cells co-expressing (A) EGFP-tagged MBD-VVCA and mCherry-actin as control or (B) EGFP-tagged MBD and mCherry-actin or mCherry-p16B as indicated. Note that MBD-VVCA and actin co-localize, whereas no overlap of MBD with actin or MBD with p16B is visible. Bar, 10 µm. (C) Phalloidin staining (blue in merge) and immunolabeling with anti-α-tubulin antibodies (red in merge) of a cell transfected with EGFP-VVCA (green in merge). Merge corresponds to boxed regions in left panels. Since VVCA does not target to microtubules, they are completely devoid of actin filaments. Bar, 10 µm. (D) Immunoblot confirming expression of EGFP-tagged MBD and MBD-VVCA at appropriate molecular weights. (TIF) [file pone.0019931.s001.tif]

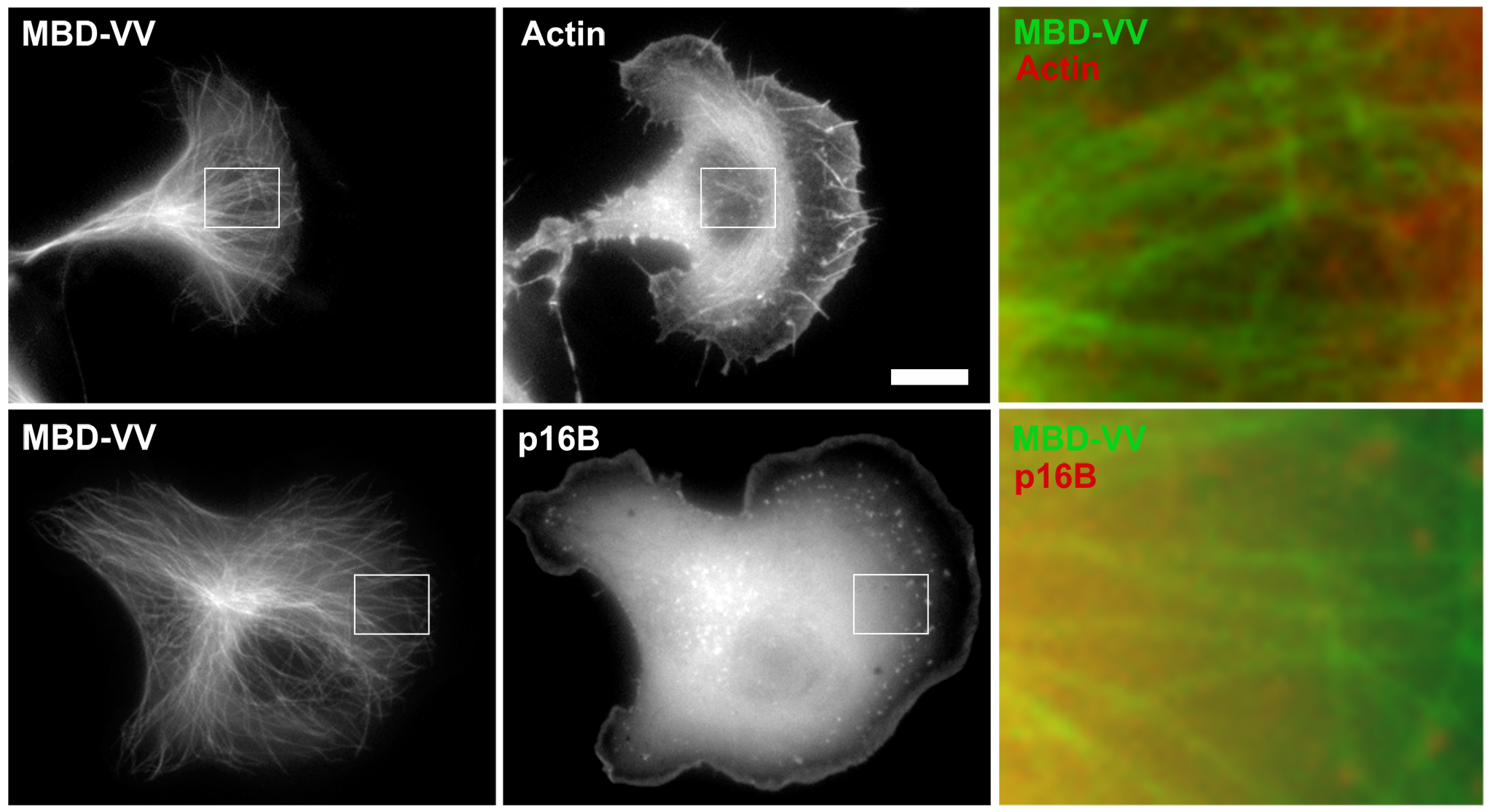

Supplement: Figure S2 — The WH2-domains of N-WASP cannot nucleate actin filaments on microtubules. Selected frames from time-lapse movie of B16-F1 cell co-expressing EGFP-tagged MBD-VV and mCherry-actin (upper panel) or mCherry-p16B (lower panel). Insets on the left are magnified in merged images on the right, revealing the absence of co-localization of MBD-VV (green in merge) with actin or Arp2/3 complex (red in merges). Bar, 10 µm. (TIF) [file pone.0019931.s002.tif]

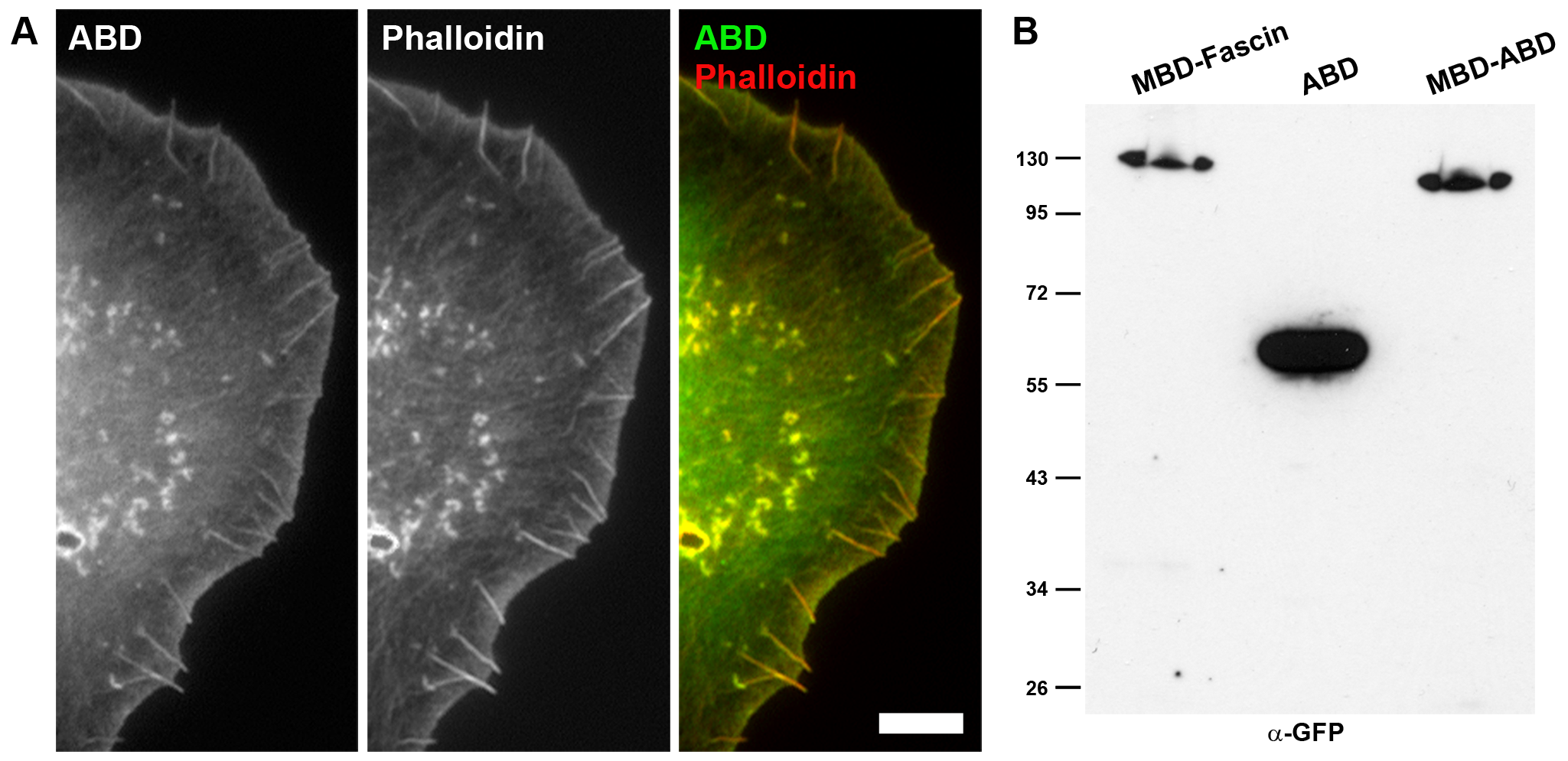

Supplement: Figure S3 — Subcellular localization of the actin-binding domain of α-actinin (ABD) and its expression compared to MBD-tagged ABD and fascin. (A) Phalloidin staining of a B16-F1 cell ectopically expressing EGFP-ABD revealing that the ABD of α-actinin robustly associates with actin networks and bundles located in the lamellipodium and the lamella behind, as expected. Bar, 5 µm. (B) Verification of correct expression of EGFP-tagged MBD-fascin, ABD and MBD-ABD as indicated. (TIF) [file pone.0019931.s003.tif]

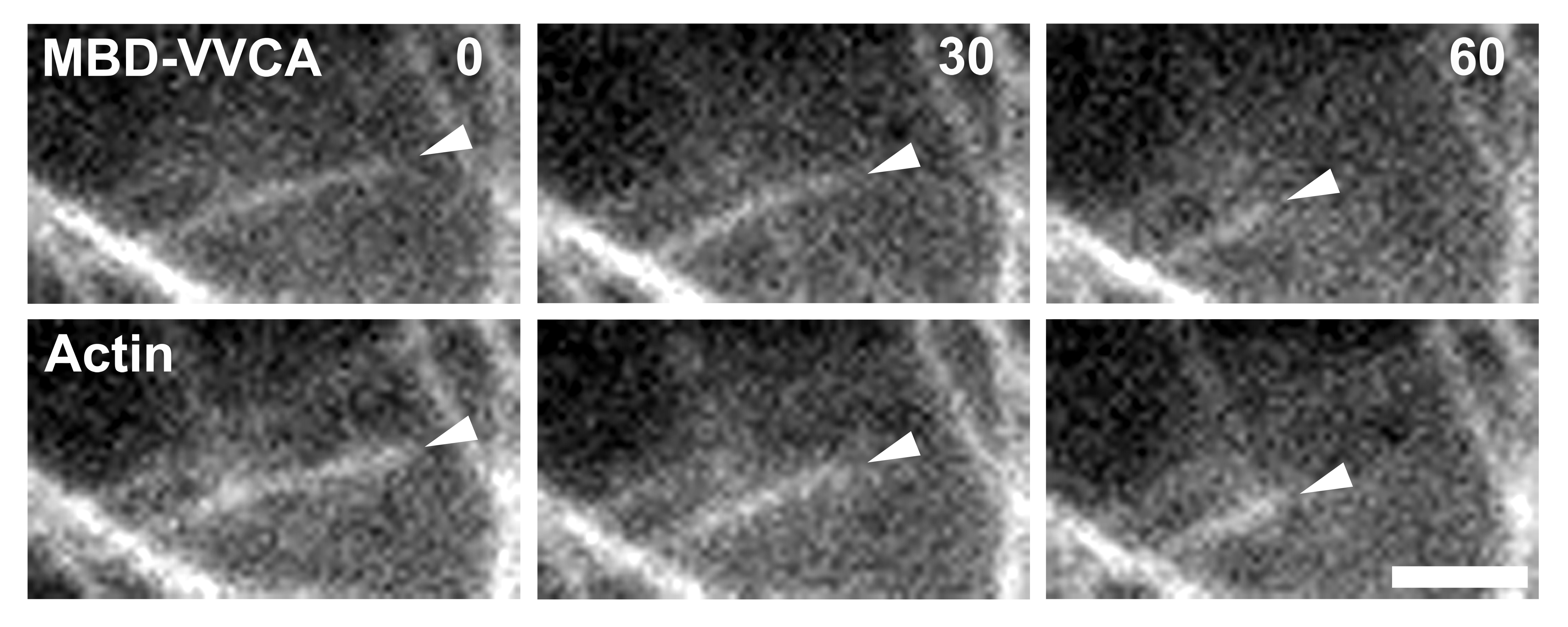

Supplement: Figure S4 — MBD-VVCA and polymerized actin dissociate instantly from shrinking microtubules. Selected frames from time-lapse movie of B16-F1 cell co-expressing EGFP-tagged MBD-VVCA and mCherry-actin as indicated. Arrowheads point to a shrinking microtubule. Time is in seconds; bar, 2 µm. (TIF) [file pone.0019931.s004.tif]

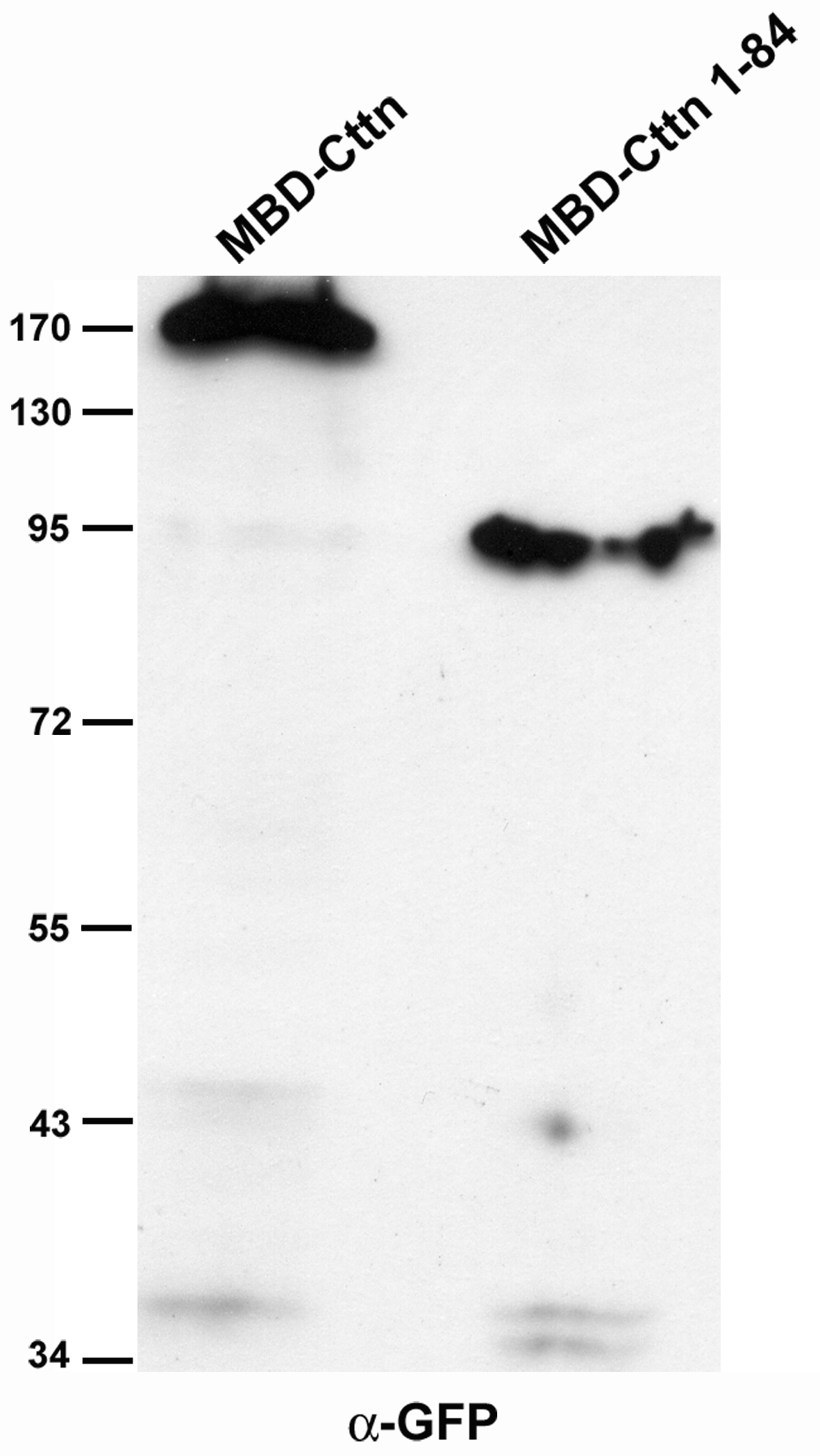

Supplement: Figure S5 — Expression control of MBD-Cttn and MBD-Cttn 1–84. Expression of EGFP-tagged MBD-Cortactin (MBD-Cttn) and its N-terminal 84 amino acids (MBD-Cttn 1–84) as verified by immunoblotting. (TIF) [file pone.0019931.s005.tif]

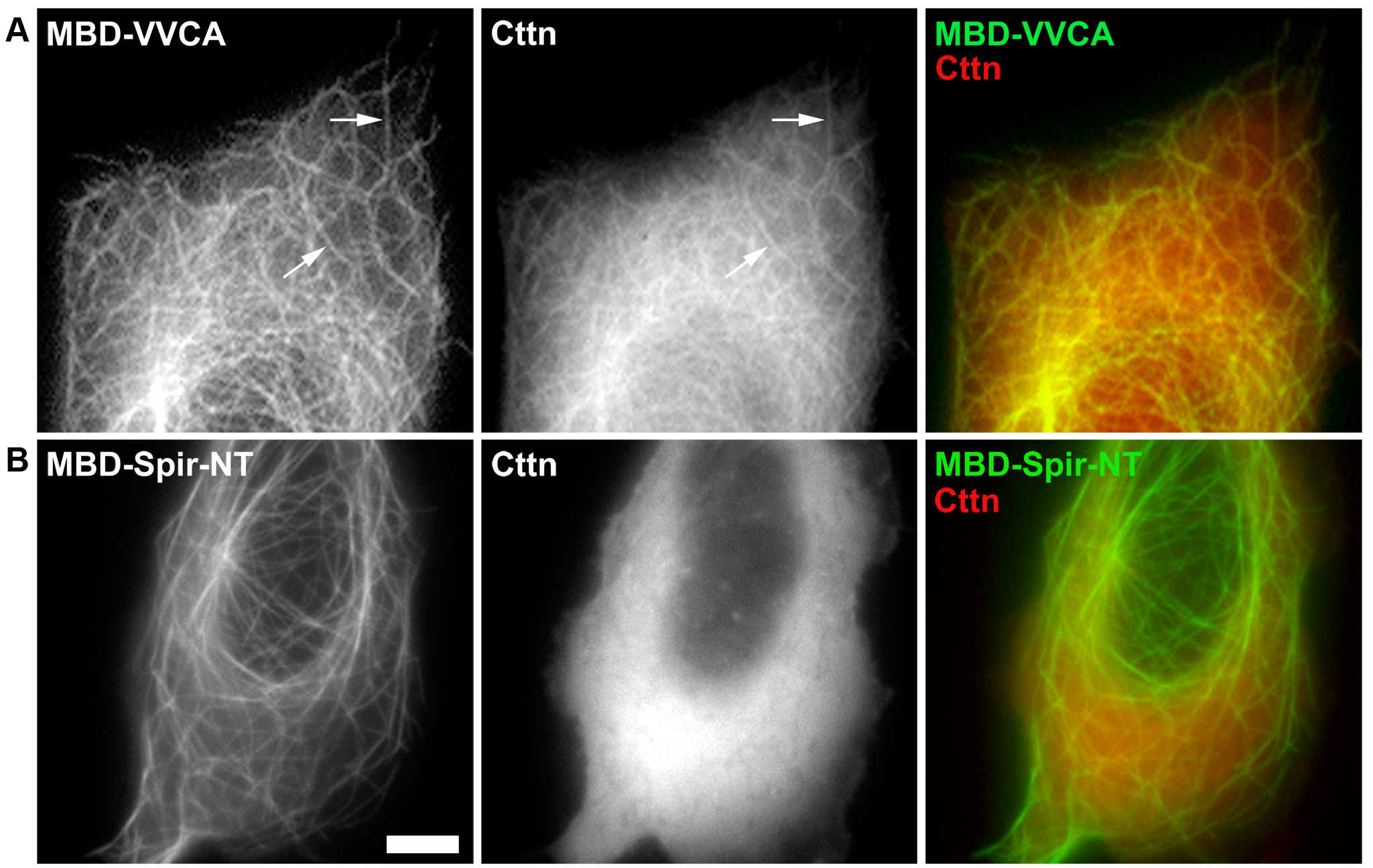

Supplement: Figure S6 — Cortactin is recruited to Arp2/3 but not Spir-NT-induced actin assemblies. Epifluorescence images of live cells co-transfected with (A) mCherry-MBD-VVCA and EGFP-cortactin (false-colored in merge for clarity) or (B) EGFP-Spir-NT and mCherry-cortactin. Merged image and arrows in (A) show significant accumulation of cortactin at MBD-VVCA-stimulated actin structures. In contrast, no targeting to microtubules decorated with Spire-NT was discernible. Bar, 5 µm. (TIF) [file pone.0019931.s006.tif]

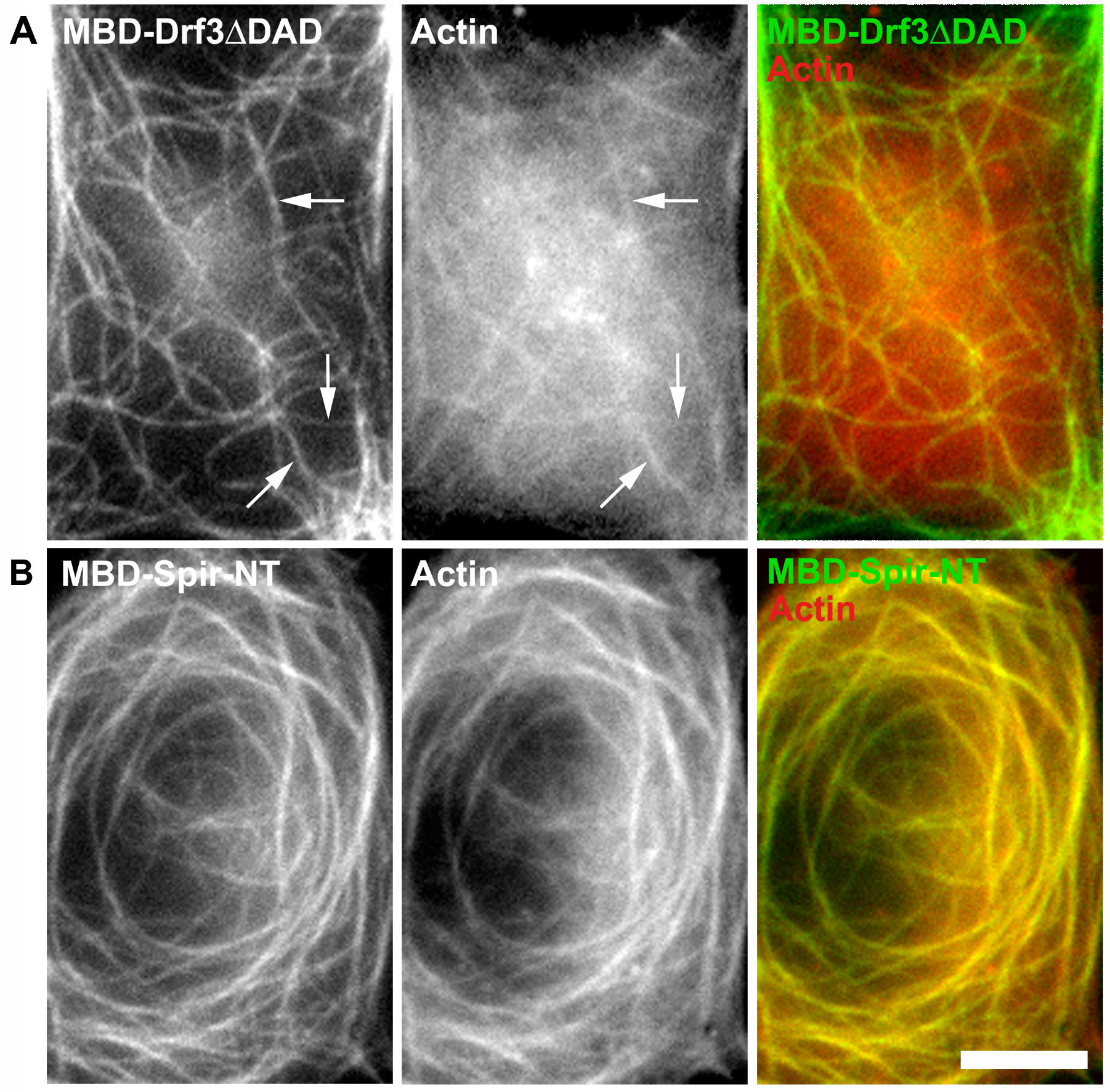

Supplement: Figure S7 — Actin polymerization on microtubules by MBD-Drf3ΔDAD and MBD-Spir-NT. Live cell imaging of B16-F1 cells co-expressing mCherry-actin and (A) EGFP-tagged MBD-Drf3ΔDAD or (B) MBD-Spir-NT. Merged images and arrows indicate robust co-localization of respective MBD-construct with actin. Bar, 5 µm. (TIF) [file pone.0019931.s007.tif]

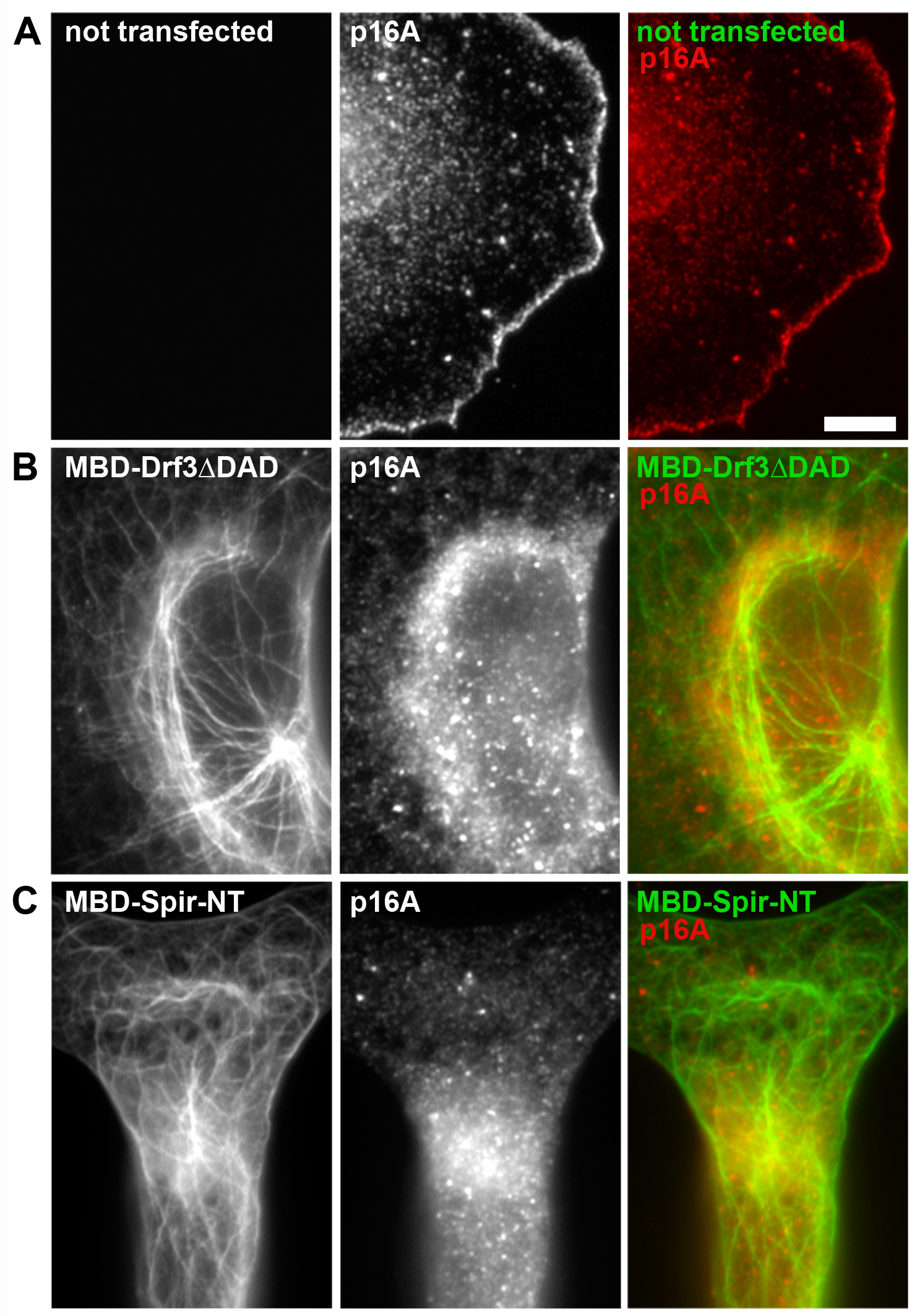

Supplement: Figure S8 — MBD-Drf3ΔDAD and MBD-Spir-NT nucleate actin filaments on microtubules independently of Arp2/3 complex. Immunolabeling experiments showing Arp2/3 complex localization (p16A) in (A) non-transfected control cell or in cells expressing EGFP-tagged (B) MBD-Drf3ΔDAD or (C) MBD-Spir-NT. Bar, 5 µm. (TIF) [file pone.0019931.s008.tif]

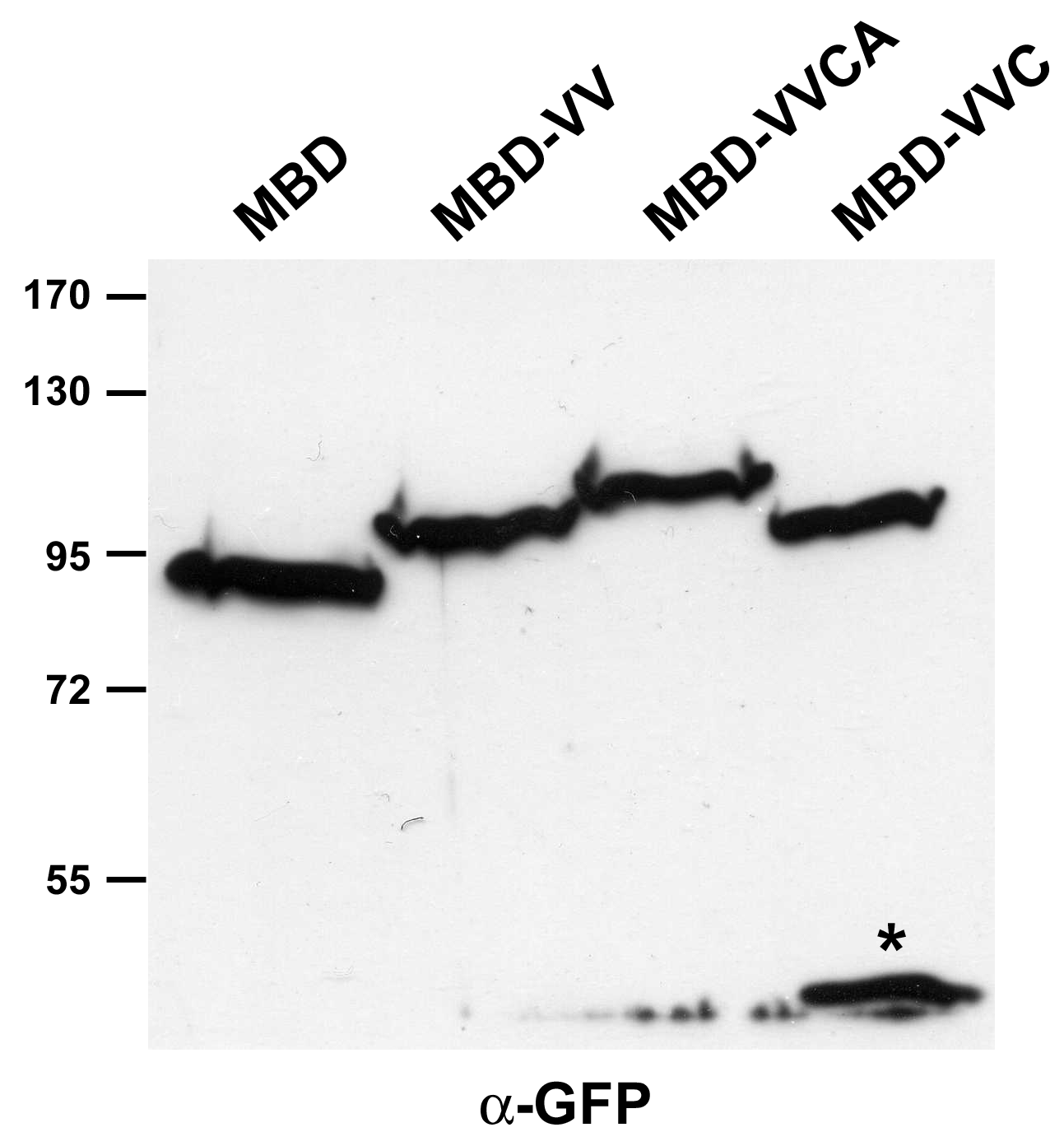

Supplement: Figure S9 — Immunoblot showing expression of EGFP-tagged MBD-VV and MBD-VVC compared to MBD and MBD-VVCA. Asterisk marks an additional, truncated product detected by anti-GFP antibodies (MBD-VVC lane) with an approximate size of 50 kDa, thus unable presumably to interfere with microtubule targeting and thus actin assembly induced by the full length fragment (Figure 1A). (TIF) [file pone.0019931.s009.tif]

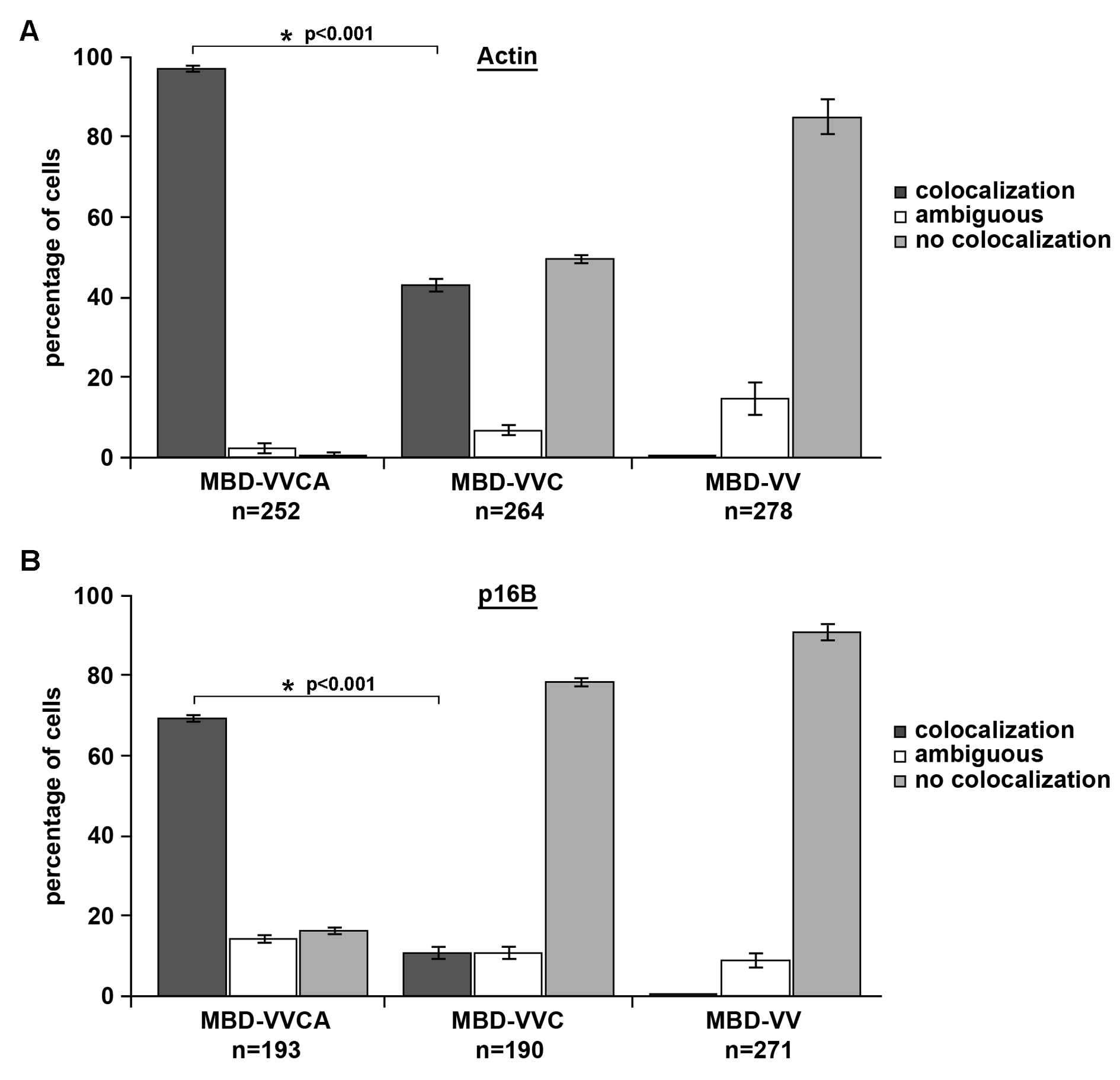

Supplement: Figure S10 — MBD-VVCA co-localizes more frequently with actin and p16B compared to MBD-VVC and MBD-VV. Cells were transfected with MBD-VVCA, MBD-VVC or MBD-VV and additionally with either actin or p16B. Living cells were classified into categories: actin (A) or p16B (B) co-localizing or not co-localizing with the respective MBD-construct on microtubules, as indicated. Cells in which actin or p16B accumulation on microtubules could not be determined due to overexpression of either construct were classified “ambiguous”. Data are means and standard errors of means (error bars) and n values correspond to number of cells analyzed. The differences between MBD-VVCA- and MBD-VVC-expressors co-localizing with actin or p16B were confirmed to be statistically significant by two-sided two-sample t test. (TIF) [file pone.0019931.s010.tif]
